# Perspectives of Additive Manufacturing in 5.0 Industry

**DOI:** 10.3390/ma18020429

**Published:** 2025-01-17

**Authors:** Dariusz Sala, Maria Richert

**Affiliations:** Management Faculty, AGH University, Gramatyka 10 Str., 30-067 Kraków, Poland; sala@agh.edu.pl

**Keywords:** 3D printing, Industry 5.0, environment, circular economy, 3D market

## Abstract

Additive manufacturing is a technology that creates objects by adding successive layers of material. The 3D method is an alternative to subtractive production, in which production involves removing material from the initial solid. 3D printing requires the initial design of the manufactured object using computer design, for example, one of the following programs: CAD, 3DCrafter, Wings 3D, Cinema 3, Blender, 3ds Max, Autodesk Inventor, and others. It is also possible to scan an existing object to be manufactured using 3D printing technology. An important element of Industry 5.0 is 3D printing technology, due to its favorable environmental orientation and production flexibility. Three-dimensional printing technology uses recycled materials such as powders. Therefore, it can be part of a circular economy, contributing to environmental protection. Additive manufacturing not only complements existing technologies by enabling rapid prototyping but also plays a fundamental role in sectors such as dentistry and medicine. This article consists of seven chapters relating to various aspects of 3D printing technology in the context of the assumptions and challenges of Industry 5.0. It examines the environmental impact and recycling potential of 3D printing technology, illustrates the economic integration of this technology within various industries, and discusses its future development prospects.

## 1. Introduction

Three-dimensional (3D) printing technology produces three-dimensional objects based on patterns previously designed on a computer. One example may be metallic objects created by applying successive layers of metallic powders melted using a laser source [1]. This new production model is consistent with the assumptions of Industry 5.0. Industry 5.0 is based on automation and digitization, which perfectly correlates with the assumptions of 3D printing technology. At the same time, however, it recognizes the need to create a people-friendly work environment that is characterized in every aspect by intuitive, interactive, and balanced interaction between technologies and people. In relation to these assumptions, 3D printing technology meets the required characteristics and is perceived as a key technology of Industry 5.0. In particular, this is due to the possibility of acceptable performance, the ability to meet environmental protection requirements, and a closed-loop economy.

**Figure 1:**
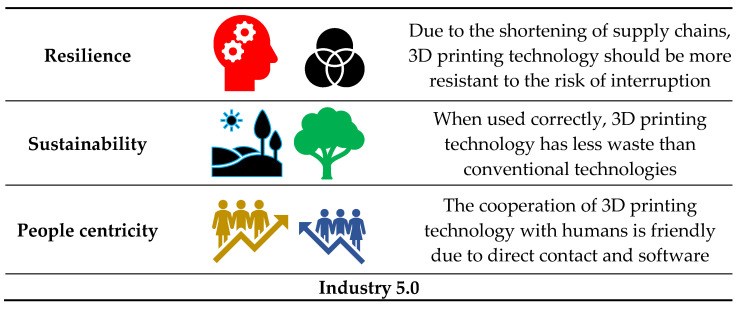
Main components of Industry 5.0.

The concept of Industry 5.0 involves the use of robotics, artificial intelligence, and the Internet of Things (IoT) [2]. Additive manufacturing fits this concept perfectly. It uses advances in computer science by initially computer modeling the manufactured object, which is then built up in layers using material deposition equipment. Industry 5.0 has three components: 1—It puts human needs at the center and recognizes the contribution of workers to the development of industry and innovation. 2—It refers to sustainable development, in particular the circular economy. 3—It focuses on resilience, understood as the flexibility of the European industry in times of crises and global changes. Each aspect of the 3D industry fits perfectly with 3D printing technology (Figure 1).

The idea of Industry 5.0 first appeared in 2016 in Japan as a response to the growing challenges of globalization, aging societies, and climate change [3,4,5]. The idea of Industry 5.0 is a harmonious cooperation between humans and advanced technologies [6]. The most advanced and important areas of activity in Industry 5.0 include the following: artificial intelligence, robotics and industrial robots 5.0, 3D printing, Internet of Things (IoT), learning, data analysis (thanks to WMS systems), and cybersecurity.

The fundamental difference between Industry 5.0 and Industry 4.0 concerns the role of humans [7]. In Industry 4.0, machines perform repetitive and routine tasks. Industry 5.0 strives to strengthen the role of employees, emphasizing the value of their skills [8]. Industry 5.0 also promotes a more plastic and personalized production process, combining human capabilities with machine performance. Table 1 shows the basic differences between Industry 4.0 and 5.0.

**Figure 2:**
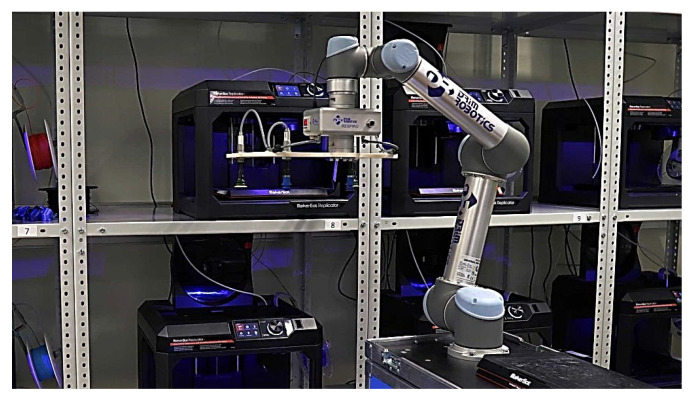
MakerBot Replicator+. Cobot’s (Universal Robot UR5 CB3 Respiro) position to automate the loading and receiving of 3D prints from printers.

Figure 2 shows MakerBot Replicator+ (Cobot purchased by Dream Robotic, Czechowice-Dziedzice, Poland), which cooperates with a 3D printer in the operation of loading and receiving prints.

Similarities between Industry 4.0 and 5.0 [9,10].

- Both Industry 4.0 and Industry 5.0 use the most advanced technologies that ensure connection and communication between machines and systems, which increases process efficiency;
- Both Industry 4.0 and Industry 5.0 use the connection of industrial systems and processes in which digitalization plays a key role;
- Production personalization is an inherent feature of Industry 4.0 and 5.0. Industry 4.0 uses flexible and modular systems. Industry 5.0 is characterized by a higher level of personalization by developing flexible and efficient production, involving the cooperation of people and robots, resulting in products tailored to individual customer needs.

Companies are paying more and more attention to cooperation between people and machines, in particular with regard to optimizing supply chain processes, increasing efficiency, and offering the highest quality products and services. In this context, an important element of the transformation towards Industry 5.0 is the increasingly easier and more efficient cooperation between employees and robots and machines [11].

One of the most important applications of 3D printing in industries is creating prototypes [12]. Three-dimensional printing is also used in the production of final elements on production lines. In some cases, especially when small series or components with complex shapes are needed, 3D printing can be more profitable than traditional production methods such as machining or casting.

According to The Economist, the symbol of the fourth industrial revolution is 3D printing, which allows you to “print” objects in cheaper ways compared to conventional methods, which require preparing molds, casting, and processing materials. Experts predict that more and more consumer products will be manufactured through 3D printing [13].

It is also predicted that traditional mass production will coexist with new technologies for many years, especially in large companies producing huge batches of goods. The SME sector will turn to new opportunities to produce complex items in short series at lower costs. However, the presented data suggest that 3D printing technology is not and will not be dominant in the economy [14]. It is rather a complementary and in many cases niche technology. However, it plays a very important role in the production system and provides essential support in the delivery of scarce parts and products.

Industry 4.0 integrates devices powered by algorithms, the Internet of Things, cloud computing, and technological platforms using artificial intelligence into a common production system and aims to increase efficiency. In Industry 5.0, humans occupy a more significant place. In Industry 5.0, cooperation between humans and artificial intelligence is preferred. Industry 5.0 is based on the use of renewable energy, waste elimination, and a circular economy. Three-dimensional printing is one of the key technologies in Industry 4.0 and 5.0 [15,16,17]. Three-dimensional printing is capable of producing any type of personalized items without expensive molds and production tools, which reduces material waste and human intervention. This corresponds to the assumptions of Industry 5.0, which places strong emphasis on environmental protection.

The benefits of Manufacturing Process Automation 5.0 include the following: reducing production costs, increasing productivity, improving product quality, reducing the risk of errors, making better use of resources, and increasing the competitiveness of businesses [18].

An undoubted consequence of automation will be the need to eliminate many jobs [19]. The consequence of increasingly widespread automation, the use of artificial intelligence (AI), and robotization will be the increasingly reduced role of humans in the economy and the elimination of millions of jobs [20]. In Germany, almost 8 million jobs could be lost by 2025 [21]. The percentage of qualified employees in this group is over 60%, so not only jobs that do not require high qualifications are eliminated. The study predicts the loss of about 1.3 million jobs in the manufacturing industry. However, as a result, better control over product quality and the reduction of raw material waste through intelligent manufacturing systems in Industry 5.0 can achieve greater competitiveness in the market and increase profits.

In the manufacturing industry, one of the most fundamental challenges will be resource scarcity [22,23]. There are already big problems with component supply chains [24]. Eventually, there will be a shortage of rare earth minerals and precious metals used in components [25]. Access to energy sources may also be a problem [26]. It would be important to create and develop technology that would enable the complete recycling of devices and equipment or its maximum reuse. The key issues of Industry 5.0 are repairability, recyclability, and green design.

Three-dimensional printing technology fits perfectly into the idea of Industry 5.0. It meets the requirements for its assumptions. Like all technologies, apart from its undoubted advantages, it has certain limitations and is associated with significant financial outlays. However, it is worth emphasizing the undeniable advantages of 3D printing technology, which make it a key element of Industry 5.0.

Industry 5.0 definitely emphasizes the role of humans in production processes, rather than replacing them with machines [27]. Nevertheless, there is a question of what place people will occupy in an industry in which a large part of tasks will be automated and performed by machines?

The organizational model in Industry 4.0 is the heart of the so-called “smart factories” or “factories of the future”. It is presented as presenting great opportunities for industries around the world. However, it is still in its infancy and exists mostly at the prototype level. Industry 5.0 is a new vision of revolutionizing production, based on the concept of human cooperation with the most advanced technologies and related to the European Green Deal [28] and the European Industrial Strategy [29]. The changes are moving towards a greener and more digital economy.

In the context of the above-mentioned features of Industry 5.0, 3D printing technology shows features that correspond to the assumptions of this type of economy [30]. Because there is little waste with 3D printing, it is more sustainable and eco-friendlier than traditional manufacturing practices. It is human-centric, in that it brings manufacturing within reach of far more people and can easily make products that are customized to the end user. Because the supply chain is relatively short with 3D printing and it offers the promise of localized manufacturing, it is also very resilient.

Additive manufacturing is usually seen as an expensive way to make things. This mainly refers to the production of large numbers of relatively simple parts that can be produced in cheaper ways using classical methods. The high costs are mainly due to the cost of purchasing AM machines. High-end models cost several hundred thousand pounds. The UK is at the forefront of the growing 3D industry [31]. UK companies already own a large number of machines, which makes high-value manufacturing such as 3D printing less prone to offshoring compared to other manufacturing industries. As traditional supply chains are disrupted and 3D manufacturing uses recycling, it provides an excellent alternative and fills the gaps found in the supply of traditional industries. Three-dimensional products need to be further developed, especially with regard to quality, but further innovation and research provide an important platform for ever better solutions in line with the Industry 5.0 economy.

In the face of the climate crisis and the growing need to protect the environment, a new approach to industry focuses on intelligent resource management, waste minimization, and product design with their life cycle in mind. A characteristic feature of Industry 5.0 is the use of human intelligence to create an increasingly sustainable and flexible production mode [32]. It is assumed that artificial intelligence (AI) will play a fundamental role in Industry 5.0, collecting and analyzing large data sets, thanks to which it will be possible to optimize production processes [33]. Thanks to it, companies can anticipate customer needs, adapt production to changing market conditions, and increase operational efficiency. AI-based systems are able to learn from collected data, which allows for the continuous improvement of processes and elimination of waste. Three-dimensional printing technology enables on-demand production and product personalization, which is key to adapting to the individual needs of customers. Three-dimensional printing also helps reduce waste because it uses only the necessary materials for production [34]. For this reason, it fits perfectly into the assumptions of industry 5.0.

**Figure 3:**
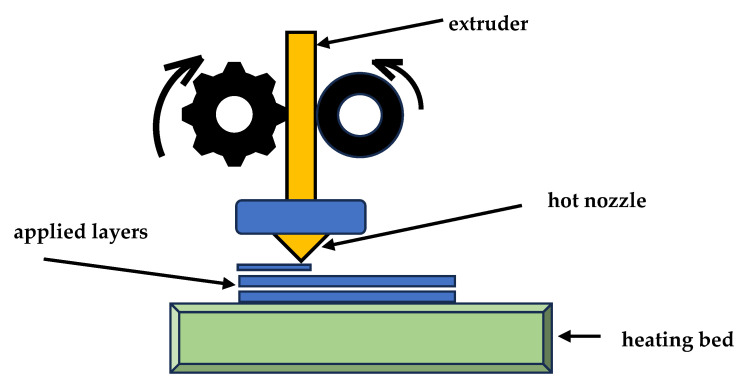
Scheme of 3D layer printing.

Knowing a specific type of 3D printing technology enables it to be used correctly. This is due to the fact that individual 3D printing technologies are dedicated to specific production areas. The most commonly used material in 3D production is metallic or plastic powder. The production process is preceded by the computer design of the product in CAD programs or other programs intended for the 3D process, such as 3D Crafter, Wings 3D, Cinema 3, Blender, 3ds Max, Autodesk Inventor, and others. Manufacturing objects involves spraying metal or plastic layer-by-layer using a nozzle until the final designed shapes are obtained (Figure 2). The share of individual 3D methods in the market varies (Figure 3). The most widespread and used technology is vat photopolymerization.

A new approach is hybrid production, which involves combining 3D printing technology with traditional production processes, such as plastic injection [35]. The interaction of these processes allows for the production of components that can be partially printed (e.g., complex internal geometries) and then finished or strengthened using traditional methods [36]. For example, it is possible to use 3D printing to create an initial shape and then refine it by injecting thermoplastics. This production system allows you to obtain a product with higher strength and better surface smoothness. The possibilities of the hybrid cooperation of 3D printing technology with various production systems were presented in the work of Saar [37] and ZainElabdeen et al. [38].

Artificial intelligence and advanced algorithms are already being introduced to create 3D models, which operate based on wide sets of input data such as texts, images, and videos [39]. Thanks to the use of AI, the process of creating 3D models is more efficient, accurate, and accessible to everyone. However, currently, only certain steps can be automated in 3D printing [40]. The human factor in this technology is essential, especially for individual tasks such as post-processing. It is believed that the prospective cooperation of artificial intelligence and 3D printing may have a positive impact on the development and improvement of 3D printing processes. In particular, the printability of an object can be evaluated and analyzed before starting any production process [41,42]. However, there are negative aspects of using artificial intelligence [43,44,45]. These include the growing dependence on artificial intelligence, which may increase vulnerability to system failures or cyber-attacks. There are also concerns that over-reliance on artificial intelligence could potentially lead to a loss of skills and a reduction in humans’ ability to solve problems on their own. It seems that the cooperation of artificial intelligence and 3D printing is a future solution that fits both the 4.0 and 5.0 Industries.

Production using 3D printing technologies applies to metals, polymers, ceramics, composites, and other materials. Conventional thermoplastics, ceramics, graphene-based materials, and metal are the materials that can be printed now by using 3D printing technology [14].

Despite various reservations and continuous improvement, 3D printing is perceived as an integral part of the fourth and fifth industrial revolutions, in which intelligent digitally controlled machines play the main role. The implementation of 3D printing speeds up the work of engineers and reduces material losses.

This technology is used to produce products from metals and polymers. Three-dimensional printing allows you to create complex and unique structures that would be too time-consuming or even impossible to produce using conventional methods.

Additive manufacturing from metals is particularly difficult due to a number of microstructural changes resulting from high temperatures [46,47]. Another challenge in this case is to control the dimensional accuracy and completion of printing the product. Moreover, compositional systems require separate development in each case.

The most commonly used technologies for metal additive manufacturing are Power Bed and Direct Deposition techniques [48]. Each method has its advantages and disadvantages, but the final choice depends on the type and purpose of the product. The great advantages of 3D printing technology for metal printing are high strength and durability of the manufactured parts, short lead times for producing prototypes and small-batch products, and lower costs compared to traditional technologies in the case of small series.

Polymers are one of the most commonly used raw materials in almost all types of 3D printing methods, except the directed energy deposition (DED) method [49]. With the rapid development of high-performance materials in 3D printing (e.g., smart polymers and advanced plastics), AM has become a fundamental process that has revolutionized the production of parts from these materials in industries [50].

Examples include shape memory polymers (SMPs), smart hydrogels, poly-liquid crystal polymers (LCPs), and liquid crystal elastomers (LCEs). Their use has led to a new production paradigm, including 4D printing, and has given the industry the opportunity to develop the production of innovative intelligent devices, robots, and biomedical products. Three-dimensional polymer printing is characterized by very high mapping accuracy, enabling you to design and print more complex designs. Compared to the mechanical processing of prototypes, 3D printing is cheaper and faster. It consumes few resources and does not harm the environment.

It should be noted that the selection of raw materials for 3D printing is limited to some extent [51,52]. It turns out that not all metals and plastics can be processed in the higher and higher levels used in 3D printing. Additionally, many of these materials cannot be recycled.

In many cases, 3D products require removing the supporting material from the structure and smoothing the surface [53]. Finishing methods for 3D products include water jet cleaning, sandblasting, chemical soaking and rinsing, air or heat drying, assembly, and more. Improperly made 3D printer models may simply fall apart.

This article contains an overview of additive manufacturing processes using metals and plastics to produce products. It presents the advantages and disadvantages of AM technology, as well as their application and development prospects for 3D printing in the context of the upcoming Industry 5.0 revolution. This article also presents and discusses selected features of 3D printing technology that determine its unique position in Industry 5.0.

This article consists of seven sections. Section 1 is an introduction to the topic of the article. Section 2, entitled “3D process classification”, presents the division of additive manufacturing methods in terms of the most commonly used processes and the division established in 2010 by the American Society for Testing and Materials (ASTM) International Committee F42. Section 3, “3D printing technology in industry”, describes the application of AM processes in different kinds of industries and illustrates the scope of the 3D printing technology market. Section 4, “Environmental aspects of using 3D printing technology”, presents the aspects in which 3D printing technology fits into the closed-loop economy. Section 5, “Prospects for the development of 3D printing technology”, presents the possibilities of AM development. Section 6, “Summary”, briefly summarizes the most important conclusions regarding the development of technology and its development prospects in Industry 5.0. Conclusions are presented in Section 7.

## 2. Definition and Mechanism of 3D Printing Process

The history of 3D printing dates back to the 19th century. In 1859, Frenchman François Willème invented a camera that made it possible to create a 3D model using several cameras. He was a photographer and sculptor [54]. In 1892, Austrian Joseph E. Blanther patented the production of relief maps. To produce them, wax boards were laminated, cut out into the desired shape, and glued together.

In this way, a 3D map consisting of several layers was created. However, these beginnings did not undergo further development of 3D printing for several decades in the 20th century. It was not until 1980 that the Japanese inventor Hideo Kodama submitted a patent application in which he described a method of hardening a photopolymer material using UV light, creating a layer-by-layer model, which is equivalent to the principle of stereolithography. Unfortunately, he was unable to pay for the patent application because his application was little known. In 1884, Frenchmen Alain le Méhauté, Olivier de Witte, and Jean-Claude André tried to obtain a patent for a process in which a liquid was hardened using a light source. They also called it stereolithography. However, the research institute they contacted was unable to recognize the potential of the invention and put the project on hold.

American Chuck W. Hull filed a patent application for stereolithography in 1981 just three weeks after the previous inventors. Three-dimensional printing technology based on his patent was first used in practice in 1983. In 1985, the first 3D design program was available, and in 1986, the inventor founded the now world-famous company 3D Systems. In 1988, the first 3D printer (SLA-1 machine) appeared on the market.

In 1992, the first selective laser sintering machine was produced at DTM [55,56], which used the process of irradiating powder with laser light. Then came a 3D printer from Z Corp that used a binder spraying process. In the late 1990s, it was possible to process metals and plastics, and further CAD programs for designing 3D objects were launched.

In 2000, additive manufacturing gained momentum and was introduced into the medical sector. It was then that a 3D-printed organ was implanted in a human for the first time. The 2000s were characterized by the further development of AM technology. Three-dimensional printers were able to produce parts for other 3D printers and found their way into workplaces.

Since 2010, new printer models have successfully printed automotive prototypes, 3D food printers, components for space stations, and jaw and bone prosthetics. Small and medium-sized companies have also benefited from 3D printing, which has enabled them to produce prototypes more cheaply. The most productive plastic additive manufacturing process is currently the Multi Jet Fusion process, in which the resulting objects have a homogeneous surface and an almost pore-free material density.

Parts or products begin as electronic files designed using computer-aided design (CAD, ZWCAD 2025) software or obtained from a digital parts repository. Then, the project file is passed through special software that divides it into slices or layers. This software, which is often unique to a given type of 3D printing or even a specific brand of 3D printer, turns slice model data into path instructions for the 3D printer to follow.

3D printing is known as additive or generative manufacturing. The idea behind this manufacturing process is to transform a numerical model into a three-dimensional model. Three-dimensional printing is the creation of physical objects from digital files. Digital data are generated by CAD modeling, data from 3D scanners, or 3D modeling. The 3D printer cannot read these data directly. Therefore, special software is required that translates the geometric shape of the product into the printer’s machine language using “G code”.

This software is called a “slicer”, and its task is to divide a 3D object into individual layers. Then, by applying individual layers, the object is built. This characteristic feature of the technology is reflected in the name—additive manufacturing. Open and closed 3D printers are produced. In the case of a closed 3D printer, the installation space is closed [57]. A 3D printer consists of a printing/heating bed, a support structure, a printed object, a nozzle, a support material, and a printing material (Figure 3).

However, the design may vary slightly within a given technology. When the printer is operating, the heating bed and nozzle first heat up. The heat bed then moves to the print head. Molten filament (special plastics, metals, or other materials) is applied to the heat bed until the first layer is completed. Once the first layer is completed, the heat bed moves down a distance of one layer in height. The second layer of filament is applied to the previous one and fused with it, and the next layers are deposited.

**Figure 4:**
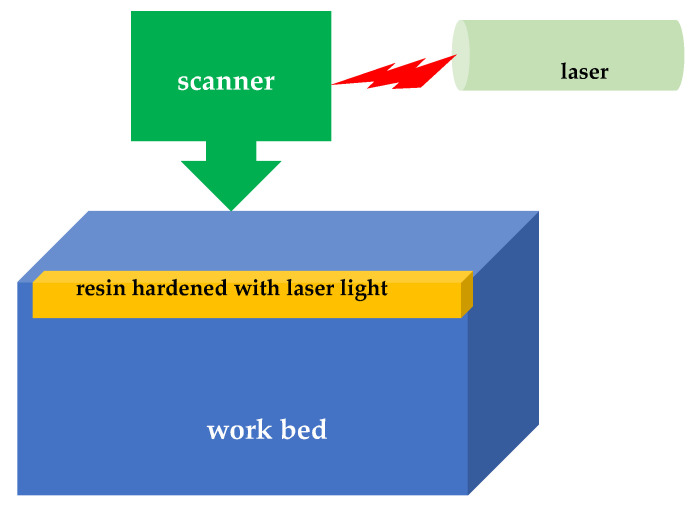
Laser melting.

Printing metal objects in the selective laser melting process involves depositing laser-melted metal powder in accordance with a programmed sequence of layers (Figure 4). In (EBM) technology, the powder is processed in the same way, only instead of a laser, an electron beam is used to melt the metal powder [58].

When using fused deposition modeling (FDM) and Fused Filament Fabrication (FFF), special plastic is first heated and then the 3D object is printed in sheets. The filament is transported via a spool to the nozzle, from which it is applied to the build plate, where it solidifies directly. The hardening of liquid plastic using UV light takes place in the stereolithography process (Standard Template Library—STL/stereolithography—SLA). The 3D object is produced in a bath of liquid plastic, and a laser is used to harden individual layers. Any support structures used are removed upon completion and the 3D object is hardened. Photosensitive plastics are used as filaments in Film Transfer Imaging (FTI). In digital light processing (DLP), a 3D object is created from a plastic bath. This process is a combination of STL and FTI 3D printing technologies.

**Figure 5:**
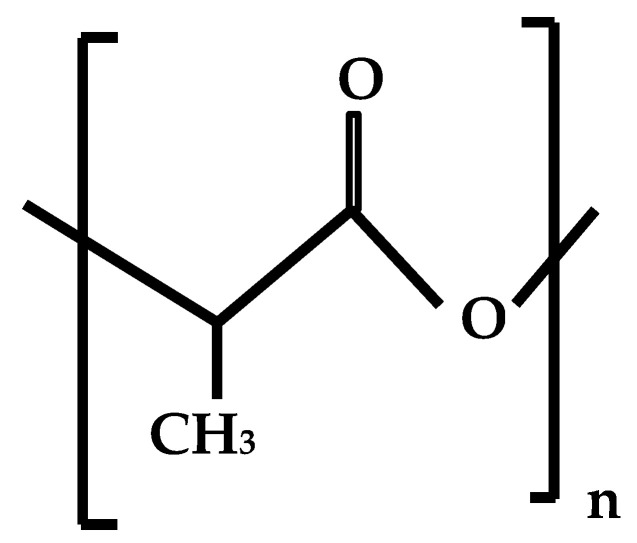
Polylactic acid, polylactide, PLA molecule. It is a polymer, bioplastic, thermoplastic polyester. Structural chemical formula (C_3_H_6_O_3_)_n_—structural pattern.

The models are characterized by a very high level of detail, and the filaments are UV-sensitive photopolymers that are hardened with light. The most important materials used in 3D printing technologies include the following:

PLA (polylactide) is one of the most popular materials for 3D printing (Figure 5). Synthetic polymers are polyesters obtained from corn starch, a renewable resource, and are biocompatible and recyclable. PLA can be processed at low melting points of 70 °C and generally retains its shape even when cooled.

ABS (acrylonitrile–butadiene–styrene) is the second most commonly used plastic in 3D printing and is also a synthetic polymer. It consists of acrylonitrile, 1,3-butadiene, and styrene. The favorable properties of this material are its strength and stiffness. It can be used for prototyping and producing final products. ABS is printed at a temperature from 220 to 250 °C and should be printed in a heated printing chamber or print bed. This allows the manufactured objects to be cooled and prevents them from deforming.

PEEK (polyether ether ketone) is a synthetic polymer from the polyether ketone group. It can be used to print objects with high elasticity and temperature resistance. It is also biocompatible and chemically resistant. Thermoplastic PEEK is approximately 70% lighter than metals with similar properties but has comparable mechanical and thermal stability. PEEK is therefore preferred by the automotive, chemical, and aerospace industries.

HIPS (high-impact polystyrene) is also a thermoplastic polymer and is produced by polymerizing polybutadiene to polysterol. It has a high impact strength and hardness and can be dissolved in chemicals. The unique properties of HIPS mean that the material is often used as a carrier material for other polymers.

PA (nylon/polyamide) has a high tensile strength, melts at 250 °C, and is non-toxic. Three-dimensional objects made of nylon are durable and resistant to damage. Nylon is unaffected by most common chemicals and is inexpensive. The disadvantage of this material, however, is that it is not suitable for private use due to its high melting points and requires both a heated printing bed and white glue to adhere to the printing bed during 3D printing.

PET (polyethylene terephthalate) is probably known to most people from drink bottles. This is also an advantage, because PET is food-safe and can be used for packaging. Because no fumes are produced during the melting process, 3D printing with PET does not require a heated printing room.

PETG (PET with glycol) achieves a high level of material transparency through modification with glycol. This also improves printing properties. This results in a lower melting point and less crystallization. PETG can be extruded faster than PET and is weather-resistant. For this reason, it is often used to make furniture and garden equipment, as well as vases.

Aluminum or aluminum alloys impress in 3D printing with their strength and good thermal properties. Three-dimensional objects are also lightweight and can be flexibly manipulated. The automotive and aerospace industries benefit from the use of aluminum alloys; engine parts, housings, molds, prototypes, air ducts, and much more are produced using 3D printing.

Titanium or titanium alloys are some of the most famous metals in 3D printing. They are characterized by exceptional mechanical properties and low specific weight. These materials are corrosion-resistant and can be used in many high-technical environments, such as aviation. Medical devices, spare parts, functional prototypes, or final parts are the most common 3D objects made of titanium alloys.

Stainless steel/stainless steel alloys are low in carbon and have high corrosion resistance. Components produced in this way also have excellent strength, good thermal properties, and high ductility. Stainless steel 3D printing is preferred for machine components or food-safe applications.

The materials used in 3D printing, in addition to plastics and metals, also include ceramics, sand, concrete, glass, and paper.

## 3. Three-Dimensional Process Classification

3D printers appeared on the technology market around the mid-1980s. They made extensive use of core technologies, including “CNC” computer control, 2D printing, and the development of lasers. During the 1990s, many of the basic principles of additive manufacturing (AM) were developed, refined, patented, and commercialized. The development of this technology is still progressing. AM was mainly used for commercial prototyping. In 2010, the basic patents for most of the original 3D printing methods expired. Thanks to this, it was possible to expand the offerings in the 3D industry, which resulted in an increase in interest in this method. The offerings of 3D printers has expanded significantly.

Printers intended for consumers and hobbyists have also become more accessible. Currently, AM is an increasingly faster and more reliable technology. It is a popular manufacturing technique not only for prototypes but also for commercial production. Currently, various technologically advanced parts and elements are produced using 3D printing. Three-dimensional printers are also increasing in size and are becoming more diverse for different purposes. Three-dimensional printers are also becoming more and more robotic, which brings the technology closer to the development directions most expected in Industry 5.0. Hybrid machines are also starting to appear. Three-dimensional printing simulation and control software is also becoming more and more mature. There has been a significant development of different varieties of 3D printing technology for various purposes and practical applications. However, as of today, there is no fully universal technology that is suitable for every application. Therefore, it is very important to select a 3D method for the intended production purpose.

There are over fifty different AM techniques, and they are usually classified into seven different processes in accordance with the ASTM F2792-12a: binder jetting, material jetting, material extrusion, vat photopolymerization, powder bed fusion, energy deposition, and sheet lamination [59] (Table 2). The advantages and disadvantages of 3D printing technology are presented in Table 3.

In 2010, the American Society for Testing and Materials (ASTM) International Committee F42 created a set of standards that were used to classify additive manufacturing processes, which resulted in their division into seven main categories [67,68]. They are as follows:

1. Vat Photopolymerization (VP);
2. Material Jetting (MJ);
3. Binder Jetting (BJT);
4. Materials Extrusion (FDM);
5. Powder Bed Fusion (PBF);
6. Laminating Sheets;
7. Directed Energy Deposition (DED).

Organizing the classification of processes allowed them to be grouped according to the method of material deposition.

**Figure 6:**
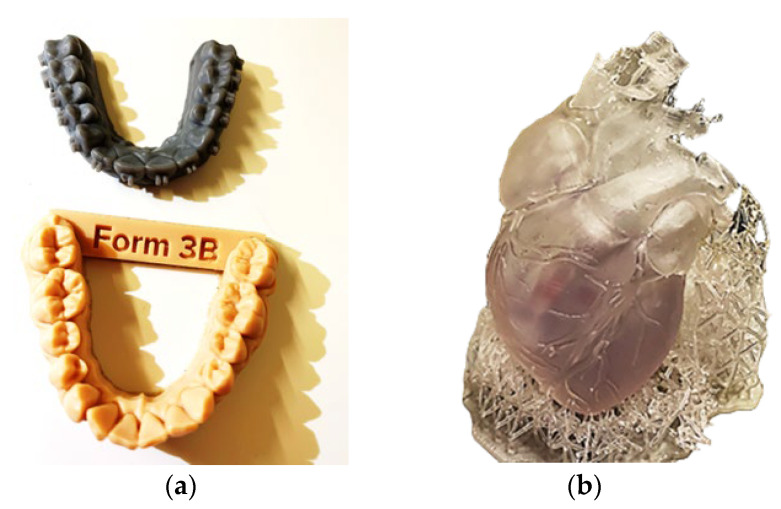
The examples of SLA products, (**a**) jaw model (**b**) heart model.

Vat Photopolymerization. This method is an over 30-year-old 3D printing method used to create a wide range of products, from prototypes to functional industrial parts. In 1986, Chuck Hull, the co-founder of 3D Systems, invented stereolithography, considered the first rapid prototyping system. Vat photopolymerization is used in 3D manufacturing to create components that can have a variety of physical and mechanical properties [69]. The most common types of vat photopolymerization are as follows: stereolithography (SLA), digital light processing (DLP), Continuous Liquid Interface Production (CLIP) by carbon, and Daylight Polymer Printing (DPP) by Photocentric and Liquid Crystal Displays (LCDs). As software, light sources, and optical transparency continue to improve, more and more types of photopolymerization are being developed [70]. Polymerization uses a vat with a liquid photopolymer resin. Ultraviolet (UV) light is used to harden or harden the resin. The platform on which the piece is placed moves the manufactured item down as each new layer hardens. Vat photopolymerization is known for its exceptionally good resolution and accuracy. The resolution in most printers is given in the XY plane. When using a laser-based system, this is the width of the laser. The layer thickness is the resolution in the Z direction. In this method, materials used are initially liquid, and the materials harden when the liquid is exposed to ultraviolet light [71]. Photopolymerization is suitable for making a premium product with good details and a high quality surface. Examples of 3D printing technologies by using photopolymerization are stereolithography (SLA) and digital light processing (DLP) (Figure 6).

**Figure 7:**
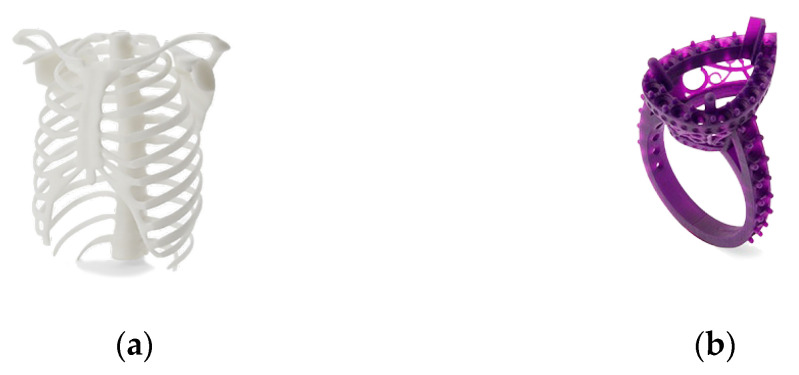
Examples of SLA products, (**a**) model of ribs and spine (**b**) ring model.

Stereolithography applies to the production of objects in liquid or semi-liquid form [72]. A part of the device, called a scraper, applies a layer of liquid to the surface, which is then hardened with a laser. Currently, more than 75% of applications in dentistry use this technology [72]. Stereolithography produces products with excellent precision and is relatively cheap. Currently, a photo stereolithography technique called the fourth generation has been developed [73]. The starting material (conventional epoxy, acrylate resins, or thermoplastic elastomers) for SLA technology is most often in the form of liquids in a tank, and from there, it is directed to the device that produces the objects [74]. This makes it easy to use SLA in industry. In the photocuring process, photosensitive resin, conventional epoxy resins, acrylic resins, cross-linked resins, and thermoplastic elastomers are used [75]. The low viscosity of the materials makes it easy to spread them smoothly [76]. Examples of SLA products are shown in Figure 7.

Directed Energy Deposition (DED). This technique covers 16% of the metal production market using AM technology. Directed energy deposition (DED) is a more complex printing process that is commonly used to repair or add additional material to existing components [77]. DED technology ensures good quality products thanks to the ability to control the structure of products. In the DED process, the dispensing nozzle is not attached to a specific axis and can move in multiple directions. The technology is suitable for the rapid production of large, high-quality metal parts at cost-effective costs. DED methods can be divided according to the energy source used. The cold spray (CS) variety uses a cold spray [78]. The material is added to the manufactured product in the form of fine particles with sufficient kinetic energy to form a dense coating or layer. The Laser Metal Deposition (LMD) method [79] uses a laser beam, electron beam, plasma, or electric arc. The feed material, which is wire or powder, is selectively melted and successively added to the working platform. DED technologies are used primarily to produce metal elements. DED processes can easily fabricate a heterogeneous material with desired properties and characteristics via successive and simultaneous depositions of different materials. In addition, a hybrid process combining DED with different manufacturing processes can be conveniently developed [80]. Directed energy deposition was developed by Sandia National Laboratories in 1995 under the name of LENS (Laser Engineering Net Shape) and then was commercialized by Optomec Design Company [81,82,83]. DED uses a highly focused beam of energy to instantly liquefy materials deposited on the substrate, creating a liquid pool, making the process similar to welding. This unique surface modification ability enables surface coating, repair, and even retrofit work on existing components that may constitute a substrate.

Binder Jetting (BJT). This technique was invented at Massachusetts Institute of Technology (MIT) and patented by Emanuela Sachsa in 1993 [84]. It is a powder-based 3D printing process. The powder is bound into 3D printing structures according to the designed shape. It is often used in the medical industry. Binder jetting (*BJT/BJ3DP*) is a rapid prototyping and 3D printing process in which a liquid binding agent is selectively deposited to join powder particles. The binder jetting technology uses a jet chemical binder onto the spread powder to form the layer [85,86]. BJ3DP is a process in which a liquid binder is sprayed onto layers of powdered materials; the materials are selectively combined, and then the product is thickened. The BJT process was developed in the early 1990s. It is a process in which a binder is printed onto a powder bed to form partial cross-sections. This concept can be compared to powder bed fusion (PBF), where a laser melts powder particles to define a partial cross-section. Several companies have adopted the process for the fabrication of metal parts. Among AM technologies, spraying binders with this method seems to be particularly promising due to the possibility of quickly producing complex structures with isotropic properties. BJ3DP machines are able to produce prototypes with properties similar to those achieved in traditional powder metallurgy.

**Figure 8:**
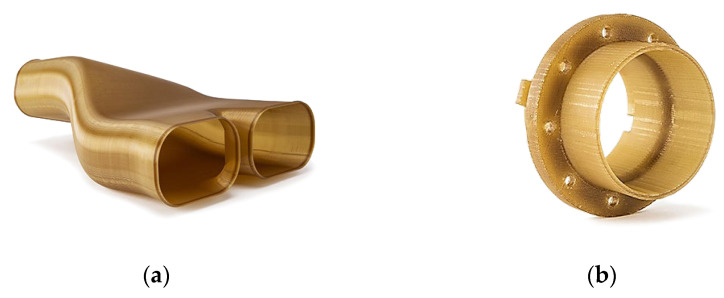
Examples of FDM products (**a**) double pipe model e.g., exhaust pipe (**b**) device element.

Material Extrusion (FDM) [14]. This technique is commonly used in the pharmaceutical industry. Material extrusion (FDM) [87,88] builds parts layer-by-layer from the bottom to the top by heating and extruding a thermoplastic filament. Next, where a support or buffering is needed, the 3D printer deposits a removable material that acts as scaffolding. For example, FDM uses a hard plastic material during the process to produce a 3D bone model. Material is extruded through a nozzle (or orifice) and selectively deposited layer-by-layer to form an object. Material extrusion was invented and patented by Scott Crump in 1989 and introduced to the market as fused deposition modeling (FDM) by Stratasys, the company Crump co-founded. Stratasys popularized the use of FDM in a variety of industries [89]. Examples of FDM products are shown in Figure 8.

Material Jetting (MJ). In this method, a printhead dispenses droplets of a photosensitive material that solidifies, building a part layer-by-layer under ultraviolet (UV) light [90]. At the same time, material jetting creates parts with a very smooth surface finish and high dimensional accuracy. Multi-material printing and a wide range of materials such as polymers, ceramics, composite, biologicals, and hybrid materials are available in material jetting.

**Figure 9:**
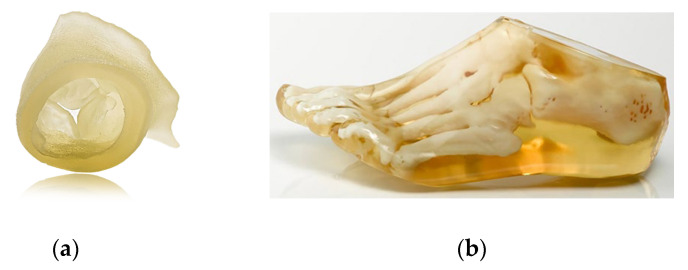
Examples of Poly Jet products (**a**) valve model (**b**) foot model.

PolyJet. This is a technology that processes liquid polymers [91,92]. A characteristic feature of PolyJet technology, unlike other rapid prototyping methods, is the ability to apply material continuously from different groups of printing heads. PolyJet technology solutions enable the control of the amount of materials issued during the process. The printing process itself is relatively simple and works based on the principles of 2D printing. Each of the applied layers of the material is fully cross-linked and exposed, unlike other methods using liquid photopolymers (SLA, DLP). There is no need to re-expose the element after printing. PolyJet technology, due to its ability to build elements from very thin layers of material with a height of 16–32 μm, is considered to be one of the most precise and accurate of all additive methods [93]. Poly Jet technology creates very precise models with a smooth surface and high dimensional accuracy. Elements created with this technology cover a wide range of materials and arbitrary shapes of the manufactured products. Examples of PolyJet products are shown in Figure 9.

**Figure 10:**
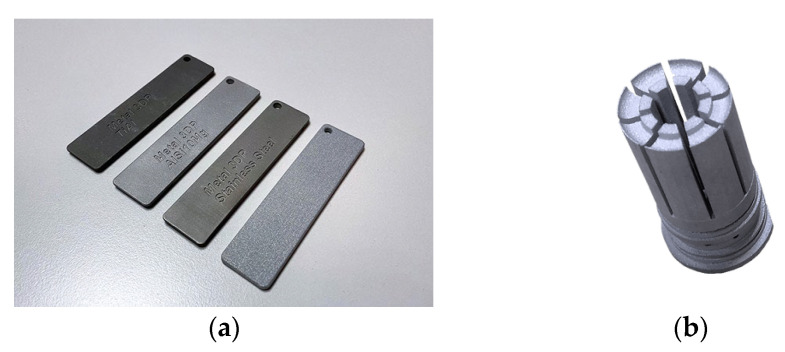
Examples of direct metal laser sintering (DMLS) products (**a**) metal tiles. (**b**) tubular model of the device.

Powder Bed Fusion (PBF). With PBF technology, it is possible to make objects with very complex geometries, internal channels, or very small parts, thus giving the finished piece a truly functional design [94]. The powder bed fusion process includes electron beam melting (EBM), selective laser sintering (SLS), and selective heat sintering (SHS) printing techniques. This method uses either an electron beam or laser to melt or fuse the material powder together. Powder bed fusion (PBF) is also known as selective laser melting (SLM), and direct energy deposition (DED) is also known as Laser Metal Deposition (LMD). This process uses either a laser or electron beam as the heat energy sources for the irradiation, fusion, and melting of powder particles. The most commonly used heat sources in PBF are various types of lasers. Examples of direct metal laser sintering (DMLS) products are shown in the Figure 10.

**Figure 11:**
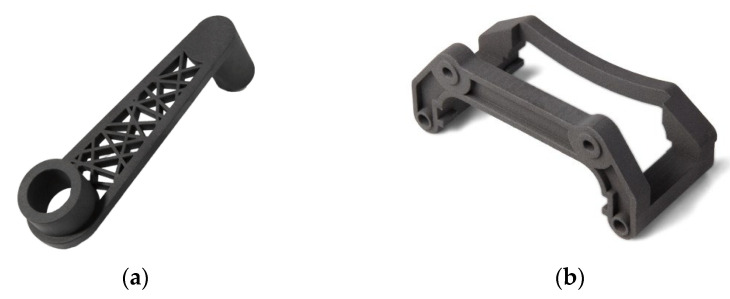
The examples of SLS products (**a**) device arm model (**b**) model of the device part.

Selective laser sintering (SLS). This technique involves the layered laser sintering of a polymer or metal powder, which gradually creates a three-dimensional print. This is one of the 3D printing techniques belonging to the powder bed category, just like direct metal laser sintering (DMLS) or electron beam melting (EBM) [95]. Thermal energy generated by a focused laser beam is used to connect individual layers of created objects [96]. The successively deposited layers of powder usually have a thickness of 20–150 µm. The big advantage of the SLS system is the stiff supporting structures, which, however, if cooled too quickly, may warp as a result of material shrinkage [97,98]. The examples of SLS products are shown in Figure 11.

Direct Metal Laser Sintering (DMLS). This technique is also known as selective laser melting (SLM) and is an industrial 3D printing process for prototypes and functional metal components and parts from powdered metal alloys [99]. It allows you to print geometry and structures that are impossible to mill on a CNC machine. Using the DMLS method, even complex items can be produced in one production cycle and thus reduce production costs. In addition, it allows the production of lighter parts compared to conventional production methods. DMSL is an industrial technology used mainly in the earliest stages of production. It is used for the production of conceptual models, functional prototypes, and the production of jewelry; it is also used in electronics and in the medical industry. The main industrial sectors in which DMSL technology is used are the aviation, automotive, medical, tool, and machinery industries. One of the leading companies that provides industrial printers for DMLS technology is EOS, which has been present on the 3D market for over 30 years [100,101]. DMLS technology has revolutionized metal processing, covering a wide range of materials and applications. The process requires great skills and is demanding because it is sensitive to parameters such as laser power, scanning speed, and others [102,103]. This method is used to make products from precious metals, hard/refractory metals, superalloys, light metals/alloys, construction steels, and **stainless** steels. The observations showed a different structural structure than in conventionally made products. Observations of the structure of metal elements produced using DMLS technology showed the presence of much smaller grains compared to elements produced using conventional methods from the same materials, for example, forging or casting. For example, in the work Ashwath et al., the Al-Si-10Mg alloy obtained an ultra-fine-grained microstructure after the DMLS process [104]. The authors of the study [105] studied a nickel superalloy, Inconel 718, which was produced using direct metal laser sintering (DMLS). Tests of samples produced using the DMSL method show greater surface roughness and a greater number of surface defects than semi-finished products prepared by precision casting [106,107]. However, the products were free from internal stresses that commonly occur in metal and ceramic components manufactured using conventional methods.

Laminating Sheets or Laminated Object Manufacturing (LOM). This technology involves combining materials such as paper, plastic, or metal foil and laminating them using welding, gluing, heating, or pressure [108]. Examples of this technology are laminated object manufacturing (LOM) and ultrasonic additive manufacturing (UAM). Sheet lamination is one of the quickest additive manufacturing methods to create composite parts [66]. The method enables the relatively quick and cheap production of high-quality composite products. The LOM process was developed by a company named Helisys and in 1991 they started selling a machine that made 3D parts using rolls of paper and a CO_2_ laser [109]. A number of other processes have been developed based on sheet lamination involving other build materials and cutting strategies.

Table 4 lists the most popular methods used for printing metals and polymers.

## 4. Three-Dimensional Printing Technology in Industry

In recent years, 3D printing has been adopted for the industrial manufacturing of many functional parts and/or articles in various sectors including automotive, aerospace, dental, healthcare, architecture, jewelers, personal protective equipment, and other consumer products. Several types of 3D printing processes are currently available commercially or at the early development stage, which typically varies in the way they form plastic and metal 3D articles (or objects) [72].

3D printing technology is increasingly impacting the economy. It shortens the product development cycle and opens up new production possibilities. Introducing innovative technologies requires a deep knowledge of the materials and their properties used for 3D printing. Currently, more and more modifications are being made in traditional techniques using 3D printing [109]. Thanks to such activities, the 3D printing industry is constantly evolving, more and more modern and specialized 3D printers are being created, as well as better software, and new materials for 3D printing are being developed, including recycled ones [59,110,111].

Three-dimensional printing, in addition to its undeniable advantages, also has some disadvantages and inconveniences [112]. The advantage of 3D printing technology is the ability to cooperate and collaborate with many currently existing production techniques. This feature of 3D printing technology facilitates the faster implementation of new solutions, as introducing innovative changes is easier. In addition, the production time of products is shortened, and operating costs are reduced, among others, due to the greater efficiency and flexibility of supply chains [113,114]. Product life cycles are shortened, which increases the speed of time to market. Three-dimensional printing technology is very useful in validating manufacturing ideas and solutions before they are implemented in industrial-scale production.

**Figure 12:**
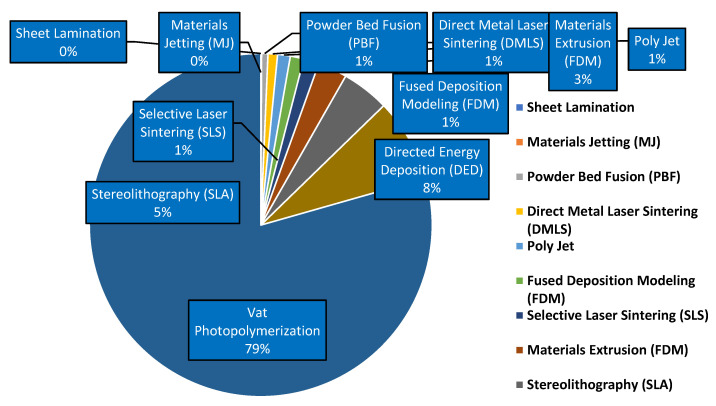
Share of 3D printing technology methods in the market.

Currently, 3D printing is widely used in the economy of almost all countries around the world. The increasing use of 3D printing is due to its flexible adaptation to the individual needs of customers. This technology also reduces the amount of waste. It is a source of non-standard solutions in various fields and areas of social and economic functioning. The market share of individual technologies is presented in Figure 12.

The most commonly used 3D printing technologies for the production of metal parts are power bed fusion (PBF) and directed energy deposition (DED) technologies [115]. There is a whole range of methods that fit into the characteristics of PBF and DED. Many manufacturers create their own solutions and introduce new names that reflect the most important features of the technology.

In the construction industry, 3D printing can be used to create component structures or even “print” entire buildings [116]. Various parts can be produced in 3D from flexible polymers [117]. Then, their inner layer is modified through chemical reactions. In this way, it is possible to produce car parts that can be adapted to changes in temperature or pressure, e.g., air in air conditioning systems or tires. Inflatable structures for temporary housing or emergency shelters can also be created [118].

Jewelry and the 3D methods for their production are presented in [119]. Jewelry is made from such metals as gold, silver, titanium, bronze, stainless steel, and the like. Powder fusion (PBF) methods such as selective laser sintering (SLS), Selective Laser Fusion (SLM), direct metal laser sintering (DMLS), Direct Metal Laser Melting (DMLM), and electron beam melting (EBM) can be used to make jewelry [80,81,82,83,120]. These methods facilitate the production of high-quality personalized jewelry from precious metals that are difficult to copy [121]. The essence of all the methods mentioned is similar and involves fusing powder particles into layers to shape the product. Three-dimensional printing enables the construction of complex shapes and structures that would be difficult or even impossible to produce using traditional techniques, such as casting or machining methods [122].

In aviation, the use of 3D printing technology has accelerated as a result of the search for increasingly innovative solutions. Due to the fact that 3D printing provides the ability to quickly design complex parts, financial outlays have been reduced and the production time has been shortened. Traditional casting and mechanical machining have certain limitations in shaping complex shapes. Moreover, they cause significant material waste during the production of parts, which amounts to up to 90% of the initial volume of the batch. Three-dimensional printing technology has the advantage of ensuring virtually the full use of the initial metal material.

Metal parts produced for the aviation industry using 3D printing technology are made of titanium alloys, nickel-based and cobalt-based superalloys, aluminum alloys, and composite materials. Despite the undoubted advantages of AM methods, one must take into account the need to control temperatures during 3D processes, the occurrence of residual stresses, as well as additional support structures for complex shapes, and attention to the quality of 3D printing. Among the various AM processes, the requirements of the aerospace industry are best met by selective laser sintering (SLS), selective laser melting (SLM), electron beam melting (EBM), and Wire-Arc additive manufacturing (WAAM) [85,86,87,88,123,124,125]. These processes can produce very compact components that can be used without any post-processing with comparable mechanical parameters and electrochemical properties to conventional ones [126].

However, the possibilities of the occurrence of structural solidification defects in 3D products cannot be ignored, such as porosity, shrinkage cavities, oxidation, etc. Research has shown that 3D-printed products may also exhibit anisotropy of mechanical properties.

Despite these types of possibilities, there are more and more applications of 3D. For example, in aerospace (SpaceX) successfully tested a 3D-printed engine called SuperDraco to be used to power and launch the escape system of the Dragon spacecraft [127]. DMLS from Inconel Super Draco was used to produce the engine compartment. SpaceX also uses AM laser sintering to produce rotors and other parts of the engines that power the Falcon 9 launch vehicle. About 100 AM parts are used in the air-cooling ducts of Super Hornet Jets [128]. Some parts of the Bell helicopter system were manufactured using AM laser sintering technology [129,130]. Bell plans to further expand the use of laser-sintered parts on its other helicopters.

Three-dimensional printing technology has also been widely used in the automotive industry. Its use has significantly revolutionized the way vehicles are produced in the modern economy. In [131], it has been shown that the current spread of 3D printing technology for the production of selected automotive and aircraft parts has improved, shortening and optimizing supply chains [132,133]. Thanks to this technology, you can easily produce some vehicle parts, such as lamps, mirror holders, dashboard elements, and others. The use of 3D printing has helped car companies secure the mass production of vehicles by avoiding unforeseen risks.

The advantages of AM technology have resulted in the creation of a trillion-dollar 3D market, which is currently developing most dynamically in the United States of America.

The global 3D printing market size reached USD 24.0 billion in 2023. As per the analysis by IMARC Group, the top 3D printing companies are increasingly focusing on various research and development (R&D) activities to develop complex and differentiated products [134]. For instance, prosthetic parts with biological materials, including bone, skin, and cartilage, for use in the healthcare industry have been produced. In addition, key manufacturers are introducing metal 3D printing technologies, such as binder jetting used in manufacturing light aircraft structures, frames, and parts, which reduce the overall time and production costs. On account of these factors, the market to reach USD 117.3 billion by 2032, exhibiting a growth rate (CAGR) of 18.7% during 2024–2032.

The top industrial plants using 3D printing technology include Stratasys Limited (Izrael, 1 Holtzman St. Science Park, P.O. Box 2496 Rehovot 7612401), 3D Systems, Inc. (Rock Hill, SC, USA), Materialise NV (Belgium Technologielaan 15 3001 Leuven), EOS GmbH (Krailling, Germany), General Electric Company (1 Neumann Way, Cincinnati, OH 45215, USA), The ExOne Company (Desktop Metal Inc.) (USA, 127 Industry Blvd, Irwin, PA 15642, USA), Voxeljet AG (Friedberg, Germany), Hewlett Packard Enterprise Company (940 N McCarthy Blvd, Milpitas, CA 95035-5128, USA), SLM Solutions Group AG (Lubeck, Germany), Proto Labs, Inc. (1099 Highland, Ann Arbor, MI 48108, USA), Optomec, Inc. (3911 Singer Blvd NE, Albuquerque, NM 87109, USA), Ultimaker BV (Utrecht, The Netherlands), Renishaw Plc (Wotton-under-Edge, UK), Beijing Tiertime Technology Corporation Limited (Beijing, China), and XYZprinting, Inc. (Taiwan, China).

There are several segments in the European 3D printing market. There is a division of the market based on components (equipment, services). The next division results from the type of technology used [46,132]. One of the countries with a significant share in 3D printing technology is Germany, which influences the development of the market in this field.

In 2024, the Europe 3D printing market size is expected to reach USD 6 billion. The prepared reports assess the prospects for the development of 3D printing technology in individual regions of Europe [135]. The analysis shows that despite strong overall development, 3D printing technology is still at an early stage of development in small and medium-sized enterprises. This indicates a rather limited, piecemeal use of 3D printing technology, concentrated in specific regions of Europe. The use of AM in industry is limited to specific areas of Western Europe. In Eastern Europe, only a limited number of 3D printer manufacturers and specialized service providers could be identified.

**Figure 13:**
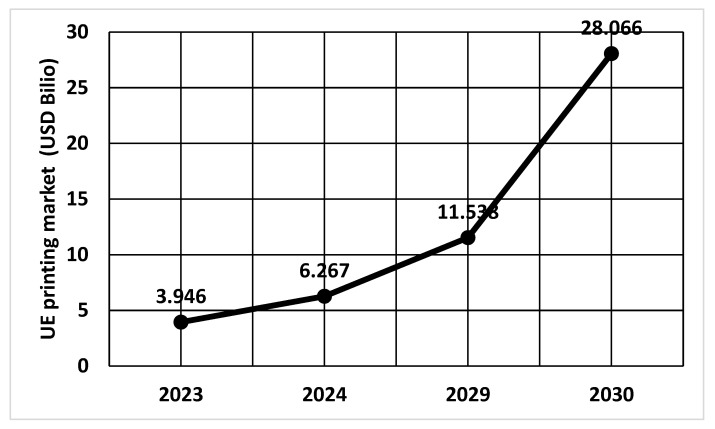
European printing market forecast.

The European 3D printing market generated a revenue of USD 6267.1 million in 2023 [136] (Figure 13). The 3D printing market in Europe is expected to reach a projected revenue of USD 28,066.9 million by 2030. A compound annual growth rate of 23.9% is expected of Europe’s 3D printing market from 2024 to 2030 [135]. The global 3D printing market generated a revenue of USD 20,369.7 million in 2023 and is expected to reach USD 88,281.3 million by 2030.

The 3D printing market in the United States is expected to reach a projected revenue of USD 16,483.3 million by 2030. A compound annual growth rate of 21.3% is expected of the United States 3D printing market from 2024 to 2030 [137].

Forecasts for the growth of 3D production in the United States of America market vary depending on the source of information. However, all of them predict a significant increase in this production in the period from 2024 to 2032.

The United States 3D printing market size reached USD 5.5 Billion in 2023. Looking forward, IMARC Group expects the market to reach USD 31.0 Billion by 2032, exhibiting a growth rate (CAGR) of 21.17% during 2024–2032. The North America 3D printing market size is expected to reach USD 16.59 Billion by 2032 [138]. United States 3D printing market size was valued at USD 3.6 Billion in 2022. The US 3D printing market industry is projected to grow from USD 4.2 Billion in 2023 to USD 13.7 Billion by 2032, exhibiting a compound annual growth rate (CAGR) of 16.00% during the forecast period (2024–2032) [139] (MRFR Database and Analyst Review).

PRNewswire (Chicago, April 22, 2024) reports that the 3D printing market is expected to grow from $17.5 billion in 2024 to $37.4 billion in 2029; it is expected to grow at a compound annual growth rate (CAGR) of 16.4% from 2024 to 2029 according to a new report by Marketsand Markets™ [140]. The growth of the 3D printing market is driven by ease in the advancement of customized products; reduction in manufacturing cost and process downtime; global government investment in 3D printing projects; availability of wide variety of industrial-grade 3D printing materials; and complex part manufacturing in the aerospace and defense sector. The growing need for supply chain efficiency and improvements in the production of spare parts are the main market drivers anticipated to drive the USA 3D printing market.

There is an increasingly growing demand for 3D printing technology in many sectors of the economy. The development of this technology is associated with ongoing technological progress, government initiatives favorable to 3D printing, and the widespread adoption of this technology in educational and research institutions. Three-dimensional printing is employed to create intricate components, prototypes, and even functional end-use products, propelling market growth. Furthermore, the ability to produce complex geometries, lightweight structures, and parts with enhanced performance characteristics is creating a positive market outlook. The implementation of several initiatives by the United States government to foster innovation, research, and development in the field of additive manufacturing is influencing the market growth. Moreover, the increasing support provided by federal agencies such as the National Science Foundation (NSF) and the Department of Defense (DoD) is representing another major growth-inducing factor. This includes stereolithography, selective laser sintering, electron beam melting, fused deposition modeling, laminated object manufacturing, and others. According to the report, stereolithography accounted for the largest market share.

In China, SLA or digital light processing (DLP) technologies are becoming more and more popular [141]. These 3D printing technologies are of interest to many Chinese universities and industrial enterprises. These include Huazhong University of Science and Technology, Tsinghua University, and Xi’an Jiao Tong University. Universities and research institutions are engaged in research on improving the quality, precision, and stability of materials and equipment for 3D printing, as well as the possibilities of the most beneficial introduction of this technology to the industry.

Until 2017, the largest local SLA manufacturers in China were Union Tec in Shanghai and ZRapid Tech in Suzhou. However, in addition to these institutions, there are a large number of suppliers of DLP equipment and materials. Industrial sectors in China using 3D printing technology include the following [142]:

- Aerospace Industry: This industry in China is investing heavily in the R&D of 3D printing technologies, which is driving demand for additive manufacturing.
- Automotive Industry: The utilization of 3D-printed metals in the automotive industry is influencing demand for the global 3D printing metal market.
- Pharmaceutical Industry: The pharmaceutical industry is using these printing techniques to manufacture personalized drugs and medical devices.
- Tooling Industry: The industrial sector’s demand for 3D printing tools and tooling is contributing to the growing demand for the global market.
- Construction Industry: While still a relatively small segment, the construction industry is beginning to adopt 3D printing as a way to create cement structures at lower costs.

In terms of revenue, China accounted for 6.2% of the global desktop 3D printing market in 2023. The China desktop 3D printing market generated a revenue of USD 299.4 million in 2023 and is expected to reach USD 1713.1 million by 2030 [141].

**Figure 14:**
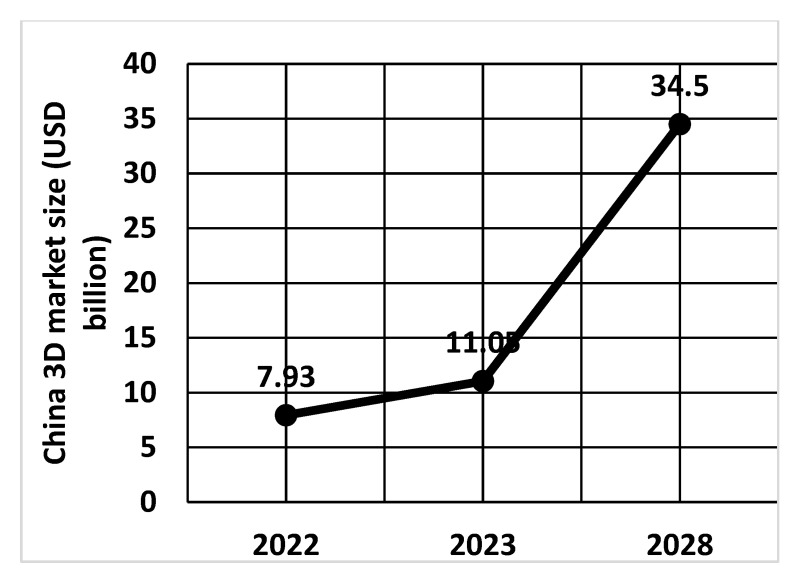
China 3D market size forecast.

The China market is expected to grow at a CAGR of 28.3% from 2024 to 2030. The expected development of the 3D market in China is shown in Figure 14. China has a 17% share of the global 3D market and ranks second in this industry.

**Figure 15:**
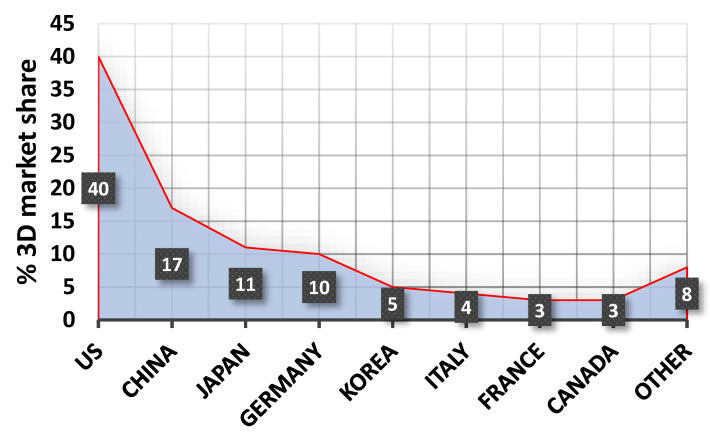
Main players, % of 3D printing market share by country.

The growth of the 3D printing market in the USA is a result of the technology’s high ability to facilitate rapid prototyping, and product development is driving the adoption of the technology in various industries, including aerospace, automotive, healthcare, and consumer goods. The global 3D printing market size was valued at USD 20.37 billion in 2023 and is expected to register a CAGR of 23.5% from 2024 to 2030 [143,144]. The Asia-Pacific region is projected to grow at the highest CAGR from 2024 to 2030. The rapid adoption of AM in the Asia-Pacific region is due to the development and manufacturing upgrades in the region. Technological developments in the automotive and medical industries, and consumer electronics manufacturing, coupled with rapid urbanization, are also contributing to the growing demand for 3D printing in the Asia-Pacific region. Currently, the three largest players in 3D printing technology are the United States of America, China, and Japan (Figure 15). The 3D printing technology parameters are presented in Table 5.

The prospects for using 3D printing as a complementary technology are determined by technological and economic barriers [145].

1. The printing speed determines whether 3D production technology can be used to complement traditional production processes. The speed of 3D printing has been considered a barrier to industrial application.
2. Dimensional accuracy is the basic indicator for measuring the quality of a manufactured product, regardless of the production method used.
3. Labor intensity is a parameter that determines the choice of technology if there are alternative production possibilities.
4. The range of materials that can be used limits production possibilities.
5. Production costs.

Despite intensive research and knowledge development, numerous publications indicate certain limitations in the use of 3D printing technology [146,147]. First of all, it is necessary to create new material solutions that meet practical needs. The next challenge is to achieve appropriate physical and chemical properties and strength properties of the manufactured elements. In addition, effective technologies for the recovery and introduction of a closed-loop economy to 3D products must be developed.

However, 3D printing offers the opportunity to reduce the need for large, centralized factories and, therefore, the opportunity to reduce the transportation, warehousing, and storage costs that come with mass production [148]. Supply chains are becoming much shorter and much more resilient, especially when using recycled materials.

The concept of producing goods closer to consumers reduces the risk of overproduction. You can print on demand based on actual needs rather than anticipated demand. As a result, excess inventory is reduced and resources spent on storage are reduced.

Economic considerations are key to introducing 3D printing into industrial activities. Three-dimensional printing is widely recognized as a valuable tool for prototyping and small-volume production [149].

Cost comparative studies have shown that for a part costing less than $20 per unit produced using 3D printing, production is only cost-effective if it replaces injection molding technology, which produces less than 200 units [150]. It follows that 3D printing is particularly suitable for small-scale production, which limits its use in continuous large-scale production.

Three-dimensional printing technologies are still, to a large extent, in the early stages of readiness and integration into industries. There is research on hybrid solutions for 3D process design and their interoperability with other manufacturing processes [151]. The combination of additive and sub-active manufacturing processes in the same work environment is called hybrid manufacturing technology [152]. The benefits of this cooperation, in particular regarding reduced material and energy consumption, improved product surface quality, increased efficiency, and ensured repeatability.

One example of the application of hybrid manufacturing is the production of prototypes and a small series of thin-walled metal components in a foundry [153]. The work concerned the creation of a vertical model from PMMA (acrylic material) for use in subsequent stages of the production of ceramic shells. Another solution proposed in the hybrid manufacturing system is a combination of conventional CNC machine production with industrial 3D printing [154]. Injection molding (IM) with additive manufacturing (AM) technologies by means of the overmolding process was presented in the work of Sampaio et al. [155].

Hybrid 3D printing defines a technology that combines additive manufacturing with subtractive manufacturing (SM) techniques. The purpose of this combination is to produce three-dimensional objects of higher quality than in the case of separate production.

The application of the hybrid method to the production of parts for the aviation industry is presented in [156]. Hybrid manufacturing, its advantages and disadvantages, and application prospects are discussed in the review paper by Sebbe et al. [157]. This new approach to production results in maximum use of the benefits resulting from the synergy of the advantages of combined technologies.

## 5. Environmental Aspects of Using 3D Printing Technology

Properly processed waste can be used as material input in 3D printing technology [158,159,160]. Reducing the high costs of input materials for 3D production is possible by using plastic waste that is easily available and has appropriate properties. This solution is also environmentally beneficial because it manages waste that is difficult to dispose of. Currently, companies providing material inputs for 3D production offer fibers made of recycled PLA or ABS [161].

Three-dimensional technology itself also produces less waste than conventional technologies, especially since the products do not need to be surface-treated. That is why 3D printing technology fits perfectly with the idea of Industry 5.0, which favors the circular economy. However, this does not mean that the carbon footprint of this technology is radically low. The production of 3D devices, their energy demand, and cooperation with computers that use critical materials lead to an increase in the value of the carbon footprint.

The production of 3D plastic products requires the use of materials that will have repeatable properties after possible recycling. Remelting used plastic parts or waste changes the primary carbon–hydrogen chains, causing them to become entangled, cracked, and cross-linked, which significantly affects the properties of the material after recycling. In the work of Ferrari et al. [162], it was found that recycled PET can be used in 3D printing technology as long as its processing produces an amorphous polymer. All resin-based 3D printing technologies, on the other hand, generate resin waste [163,164]. Three-dimensional printing technologies also generate waste. It has been determined that approximately 70% of the powder in the selective laser sintering (SLS) process may not be sintered. It is possible to recycle the powder remaining after the process. Such a recycling process was developed by Weinmann and Boten [165]. An activating agent is added to the spent powder to split the long polymer chains of the spent powder.

Plastic recycling in individual European Union countries occurs at different levels. In Poland, the recycling of plastic packaging waste covers approximately 35% of the waste mass, which is lower than the EU average of 42% [166]. Europe is the second largest producer of plastics in the world with a share of 18.5% of global production (2017). A total of 29.4% of global plastic production comes from China and another 17.7% comes from North American Free Trade Agreement (NAFTA) member countries.

The globe is littered with plastic waste [167,168]. Micro and nanoplastic particles are present in seawater, drinking water; they are practically everywhere [169,170]. Studies show that plastic particles are in fish, and through their consumption, also in human bodies [171]. A lot of evidence indicates that plastic is a common pollutant found in all organisms on Earth. The complete recycling of plastic parts and products could save the world from a bleak future of plastic pollution [172].

Metals are a much more rewarding example of the possibility of recycling and building a circular economy. Aluminum [173], steel, iron [174,175], non-ferrous metals [176], and others [177] can be almost totally recycled [178]. This creates an excellent prospect for the development of a self-sufficient economy, saving energy by eliminating the need to extract and process primary materials. A great example of practically 100% recovery is the recycling of aluminum. The secondary smelting of used metal products or post-production waste is already carried out to a large extent in many branches of the economy that obtain secondary waste metals.

The recovery of rare earth and critical metals is problematic [179,180,181]. The demand for these metals in 3D printing technology is mainly related to the need to use computers to program the technology. The growing demand for critical metals is facing barriers resulting from China’s hegemony as their main supplier [182,183]. New sources of rare and critical metals are being sought [184]. Recent reports indicate the discovery of deposits in Norway [185,186,187]. The idea of the EU is to become independent of external supply chains, which may become possible in the light of such reports. Currently, due to the global spread of supply chains, each disruption also has global negative effects.

Metal resources are limited. Therefore, the circular economy is an inevitable necessity and is one of the priorities of Industry 5.0. All studies focused on metal recovery by remelting point to several problems related to the production of alloys, with the assumed chemical composition from waste with different chemical compositions. Current technologies are good at achieving the assumed chemical composition of alloys. The energy consumption for remelting is lower than for the production of primary metals. The ore extraction stage, which consumes large energy and financial outlays, is also eliminated. The undeniable benefits of the circular economy are collapsing in areas related to metal recycling, which are difficult to isolate from manufactured products.

**Figure 16:**
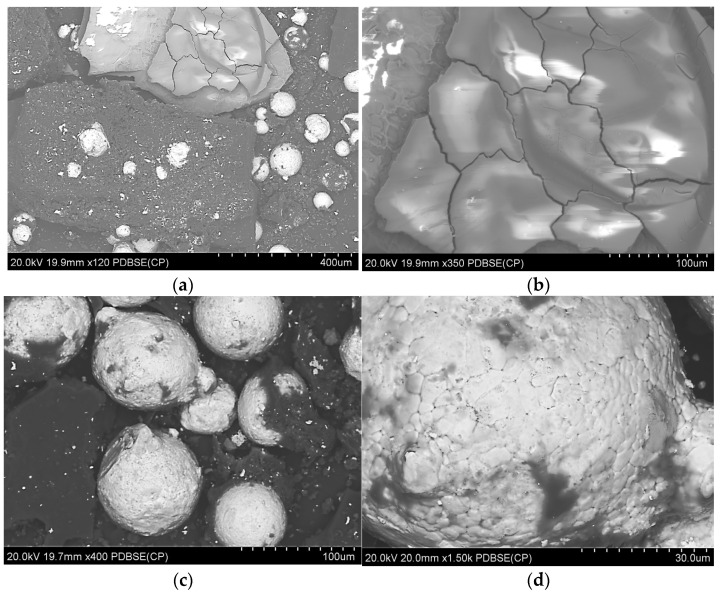
Structural defects of the WC-Co powder (scanning microscope). (**a**) Powder with different particle sizes. (**b**) Cracked surface of a large powder particle. (**c**) Spherical powder particles. (**d**) Powder surface structure.

All 3D printing technologies producing products from metallic powders (L-PBF, EB-PBF, and DED) require special attention with regard to the quality of the powders, especially recycled powders [188]. Recycled powder may contain binder residues from which it must be cleaned. Other typical powder contaminants may include moisture, oils, metal residues from the distribution system, and other foreign particles that may adversely affect the flowability, melting, and mechanical properties of the products. In particular, due to the high surface-to-volume ratio of powder particles, powders are more reactive and can absorb large amounts of moisture more easily. The higher reactivity of powders may also result in the development of internal defects in the form of voids, cracks, and other defects, in particular surface defects. The numerous occurrences of defects cause changes in the properties of powders and the need to reject them. Powder control is of great importance, especially in the production of responsible parts in medicine, aviation, transport, and other economic sectors. Powder recycling must take into account not only the possibility of contamination, but also carefully control the chemical composition of the powder, the stability of which is necessary to maintain repeatable 3D production results. It is advisable to examine the structure of powders with high resolution microscopes, such as scanning microscopes and even transmission electron microscopes (Figure 16).

As a result of the lack of technology enabling the complete recovery of secondary raw materials, as well as due to the non-recyclable additional materials in the waste, landfills are growing. To some extent, they can be treated as secondary mines, waiting for solutions to enable their full use. Human activity that processes the Earth’s crust in search of metals and other ores may stop at some point due to the depletion of resources. This threatens elements such as silver and gold, as well as other metals. Then, only the recovery of metals from used products and waste will remain.

The source of the great demand for materials is an intensive consumerist approach to the surrounding and used equipment and products. They are not repaired, but thrown away, which constantly increases the demand for various types of materials, the resources of which are becoming increasingly depleted due to intensive exploitation.

Currently, it is estimated that less than 10% of materials in the 3D printing industry are recycled [77]. Materials suitable for recycling are primarily thermoplastics. However, there are forecasts that 40–60% of 3D-printed items will be recyclable.

Recycled plastic used in 3D printing can be recycled from the 3D process or from other manufacturing processes such as injection molding [77]. It turns out that failed 3D prints constitute over 80% of the waste generated during 3D printing. Failures that are the source of failed prints can be caused by many reasons, from problems with a low filament quality and substrate adhesion to cutting errors and equipment failures.

The most popular 3D printer filaments, ABS and PLA, are not recycled. The problem is that under ASTM international resin identification codes, both ABS and PLA are classified as Type 7, or “other”, which is not typically recycled. They must be recycled specifically.

As the popularity of 3D printing increases, the problems of the disposal and waste management of plastics used in this technology are increasing.

According to current knowledge, approximately 33% of all 3D prints becomes waste. For example, in the UK, 3D fiberglass printing generates 379,000 kg of plastic waste [189]. At the end-of-life, approximately 70% of 3D printing operators do not recycle their waste. This suggests that waste from 3D printing is likely landfilled, having a harmful impact on the environment. There is also no established 3D printing waste disposal (EoL) system.

The cost of recycling, depending on the amount and density of waste, is usually on average GBP 4–5 per kilogram in the UK. Aggregate pricing can be intimidating to operators, be financially difficult, and make them reluctant to incur such expenses.

To minimize waste associated with 3D printing, it is necessary to use environmentally friendly practices such as optimizing print settings, printing multiple objects in one job, using recycled or biodegradable fibers, and designing modular components to enable their reuse [34,190]. Focusing on ways to reduce waste from 3D printing contributes to a more sustainable economy by turning waste back into usable material. Despite the reported environmental benefits of AM, it is important to note that 3D systems were not originally designed with environmental efficiency in mind. It is only now that attention has begun to be paid to the amount of 3D waste and the possibilities of its disposal. This is related to the increasing dissemination of this technology and, therefore, to an increasing amount of waste.

Systemic solutions to this problem that fit into the global circular economy system are necessary. There is also a need for further research into the environmental impact of waste from 3D printing materials. An important issue is the assessment of the possibility of waste recycling and its selection in terms of quality for reuse.

On the other hand, there is information indicating effective recycling. One of the companies that recycles materials from 3D production is Stratasys. Fred Fischer, director of materials business development at Stratasys, said that “Stratasys has a company that comes and picks up all our scrap, old part samples and construction sheets” [191].

These companies usually grind all collected materials. The material obtained after grinding is often used to produce products such as carpets, plastic terraces, garden furniture, etc. Companies that collect and recycle 3D materials exist practically everywhere in developed countries. It seems that we can expect this service to develop along with the development of 3D printing.

An example of good practice is the original project that was created in Vienna, Austria [192]. The EOOS design studio designed and constructed a 3D-printed tricycle using waste from various supermarkets in the capital! The project used 70 kg of waste, which it transformed into material for 3D printing. It was then deposited layer-by-layer to form the tricycle’s chassis. The tricycle was strong enough to accommodate two adults.

Another example of recycling related to the 3D printing industry was the printing of clam pots designed and 3D-printed by the Dutch architectural firm Aectual. In collaboration with DUS architects, both organizations decided to combine 3D printing and recycling when developing the flower pot models. In particular, 100% recycled plastic was used and transformed into production material for use in four 3D printers mounted on working arms.

The largest pot had dimensions of 98 × 40 × 29 cm, and the highest pot had dimensions of 55 × 45 × 49 cm. Hans Vermeulen, co-founder and CEO of Aectual, commented on the move as follows: “We need to get rid of the negative perception that plastic is a single-use product”, which shows the company’s interest in further involvement in 3D printing to promote recycling.

The current state of recycling is unsatisfactory. This statement does not just apply to 3D printing. However, in the light of Industry 5.0, one of the assumptions of which is environmental protection and a closed-loop economy, it is necessary to develop a systemic solution to problems related to recycling. In particular, the recycling of polymer waste materials, used parts, and machines is required.

## 6. Prospects for the Development of 3D Printing Technology

Three-dimensional printing technology is seen as an excellent example of generating little post-production waste. There are also many solutions for recycling used products. Current studies indicate that in the long term, there should be a decisive development of this technology, in particular in Industry 5.0.

The significant cost of 3D printing technology devices limits its widespread use. In the EU, the greatest development of 3D printing technology took place in Great Britain. It is not possible to replace existing technologies that are well-developed and proven with 3D printing technology. Nevertheless, 3D printing technology is and will continue to be an integral part of industrial activity. There are areas of activity in which it occupies an important place, such as dentistry [193,194]. Another area of application is the creation of casting molds [195].

By shortening production time and reducing costs, 3D printing technology influences manufacturing industries. Another advantage is also the possibility of using many different materials, such as metals, alloys, plastics, ceramics, and others. High printing speeds increase efficiency, which promotes product launches [196].

The ability to personalize products manufactured with 3D printing is another advantage of this technology, due to the growing demand for the production of non-standard products dedicated to consumers. The consequence of this trend is a significant increase in the demand for printers, especially SLA printers [197].

The stereolithography (SLA) technology 3D printing market is projected to witness a compound annual growth rate of 19.27% to grow to USD 6.746 billion by 2028, from USD 1.964 billion in 2021. Three-dimensional technology plays an important role in prototyping in manufacturing operations in sectors such as cosmetics, industrial manufacturing, and others. Creating the final version of the products requires the creation of numerous prototypes, which can best be developed in 3D printing technology. In this context, a significant increase in employment in the AM industry in the production of prototypes is expected. According to the U.S. Bureau of Labor Statistics, 1.2 million workers were employed in prototype production in 2016, representing more than a quarter of the jobs available in the manufacturing industry.

The demand for large numbers of prototypes before final production is driving the 3D printing technology market. In the US, for example, 30,000 new products are introduced to the market every year. The value of this market in the US in 2021 was USD 1.964 billion. In 2028, it is projected to reach USD 6.746 billion, with a CAGR growth rate of 19.27% from 2021 to 2028. In the case of China, the total market of the 3D printing industry in 2017 is estimated to have reached RMB 17.3 billion (approximately USD 2.7 billion, and globally, approximately USD 6.0 billion), and the industry is expected to reach RMB 35.0 billion (approximately USD 5.4 billion), global USD 21.2 billion) [92]. In China, SLA/DLP technologies are mainly used in industrial production, the production of personalized products, and in biomedical industries [198].

The Europe 3D printing market size is estimated at USD 6 billion in 2024, and is expected to reach USD 11.54 billion by 2029, growing at a CAGR of 14% during the forecast period (2024–2029). Europe is a major hub for 3D printing technology. Regarding the EU, there is a continuous growth of the 3D printing market in various sectors such as industrial products, and aerospace, automotive, defense, healthcare, education, and research industries [141,199].

The 3D printing technology market, despite high initial costs related to the implementation of new solutions, is constantly developing thanks to the benefits of non-standard products, increased efficiency, and better product quality. The use of this technology in intelligent architecture, automotive, and medical industries is expected to drive the growth of this market. In 2020, the 3D printing industry faced challenges related to the COVID-19 pandemic. After a certain slowdown in the economy in the EU, there was a reaction and anti-crisis measures. The resumption of economic activity, its further development, and the continuation of the upward trend, which also applies to the 3D printing market in Europe, are expected.

**Figure 17:**
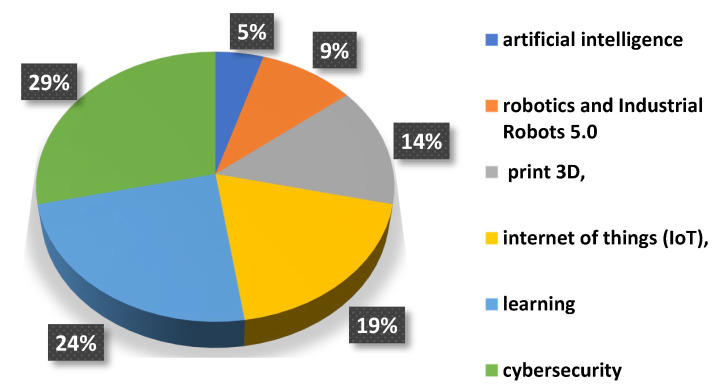
Main elements of Indusrty 5.0.

The idea of Industry 5.0 first appeared in 2016 in Japan as a response to the growing challenges of globalization, aging societies, and climate change [3,4]. One of the keynotes of Industry 5.0 is smooth cooperation between humans and advanced technologies [5]. The most advanced and important areas of activity in Industry 5.0 include the following (Figure 17):

— Artificial Intelligence;
— Robotics and Industrial Robots 5.0;
— 3D Printing;
— Internet of Things (IoT);
— Learning;
— Data Analysis (thanks to WMS systems);
— Cybersecurity.

The benefits of Manufacturing Process Automation 5.0 include the following: reducing production costs, increasing productivity, improving product quality, reducing risk of errors, making better use of resources, and increasing the competitiveness of businesses [200]. Industry 5.0, unlike 4.0, assumes greater human participation in production processes, instead of replacing it with machines [6,201].

Three-dimensional technology has undeniable advantages, including the following [3,202,203,204,205]:

- Revolutionizing supply chains by offering on-demand production of custom parts and components.
- Offering local printing, and minimizing lead time and transport costs, which results in shorter supply chains, greater stability, and risk resistance.
- Eliminating the need for large, static inventories.
- Adapting products to individual customer needs
- Minimizing waste
- Optimizing material consumption through the production of additives.
- Reducing the price of parts through increased printing speeds, although the costs of 3D machines are high.
- Leading digital transformation in the era of Industry 5.0.

However, standards need to be developed so that printers and post-processing systems can work together. Currently, such solutions are poorly developed, which hinders the functioning and development of 3D printing technology.

In addition to its undeniable advantages, 3D printing technology also has disadvantages [146,147]. They are related, among other things, to the limited scope of processed materials.

For example, it is not possible to print raw materials. It is necessary to process the manufactured parts, in particular to remove the support parts and clean them. The unit cost of parts in mass production is unprofitable. Moreover, the size of the printed parts is limited.

The greatest use of 3D printing technology is observed in the aviation and automotive industries [138,164]. Both sectors benefit from the weight savings and complex geometries offered by 3D printing. Companies like Airbus and Porsche are already using 3D-printed parts in their products, leading to more efficient designs and lighter solutions [206]. In addition to weight reduction, there is also a significant reduction in production costs, minimizing waste, and lead times for part production are shorter.

Today’s cars are perfection in engineering and design. With 3D printing processes such as selective laser sintering (SLS) and fused deposition modeling (FDM), manufacturers can test and refine designs at speeds previously unimaginable. This acceleration in the prototyping phase means faster innovations and a faster time to market.

Three-dimensional printing is widely used in medicine and dentistry. It produces various types of implants and organ replicas. Dentists can produce models of dental arches, orthodontic overlays, surgical templates, individual implant abutments, and other accessories, including products made of certified biocompatible materials that are approved for sterilization. The main advantage of 3D printing in dentistry and medicine is the ability to produce individual and personalized products [207].

AM markets are expected to have a greater degree of flexibility relative to other technologies, facilitating the introduction of more product variants becoming available [208]. If the costs required before production can be reduced, the barriers to entry will become lower. For small series and probably hybrid production, product prices should be lower. An increased variety of AM products is expected to provide consumers with the most preferred goods. The 3D printing technology market is projected to grow from USD 264.46 billion in 2024 to USD 761.35 billion by 2032, exhibiting a compound annual growth rate (CAGR) of 14.13% during the forecast period (2024–2032) [209]. In the USA, the global 3D printing materials market is projected to grow from USD 2.5 billion in 2022 to USD 7.9 billion by 2027 at a CAGR of 25.6% during the forecasted period [209]. China’s 3D printing market is expected to reach a projected revenue of USD 7707.2 million by 2030 [209]. The 3D printing market is expected to have a compound annual growth rate of 27.6% during 2024–2030. The China market is expected to grow at a CAGR of 27.5% from 2024 to 2030.

## 7. Summary

The emergence of additive manufacturing technology has changed the way products are manufactured. This technology is intended to be an alternative to classic production. In subtractive manufacturing methods, material is removed from a block to create a product. In 3D printing, the actions are the opposite. AM builds objects layer-by-layer based on digital models. In this context, it saves material because it does not produce post-production waste.

Changes resulting from an unconventional approach to the construction of objects, parts, prototypes, and other production elements are an example of innovative solutions creating prospects for a new definition of manufacturing processes. This approach is an integral part of Industry 5.0, which emphasizes the role of humans and puts emphasis on their harmonious cooperation with advanced technologies. In Industry 5.0, sustainable industrial development based on environmentally friendly technologies is important. Three-dimensional technology should become one of them.

AM encompasses a variety of technologies, each tailored to specific applications. The main 3D printing technologies are binder jetting, material jetting, material extrusion, vat photopolymerization, powder bed fusion, energy deposition, and sheet lamination (Table 2). Each mentioned technology contains different varieties adapted to specific technological solutions. Three-dimensional printing technology can be applied to various materials. However, there are material limitations resulting from high temperatures in 3D printing processes, which affect the structure of the materials and affect their final properties. Structural changes also occur as a result of the transition to the liquid state and then secondary crystallization. Polymeric materials are most often used (Table 3).

The need for surface treatment after the 3D printing process increases production costs and extends the production time of products.

Three-dimensional printing technology is perfect for unit production dedicated to specific orders. The personalization of 3D printing production meets the assumptions of Industry 5.0. One of the determinants of 3D printing technology’s belonging to Industry 4.0, and in particular to Industry 5.0, is the possibility of cooperation with artificial intelligence. Three-dimensional printing control software, coupled with artificial intelligence, creates the prospect of new technological solutions.

Three-dimensional printing technology, despite its significant advantages and revolutionary method of production, due to its features and economic aspects, in the long run can only be used as a complementary element of the production of selected industrial projects. It is definitely not suitable for use in assembly lines/line production. However, it is possible to adapt it in nest production.

There are great opportunities for 3D printing technology in medical and dental applications, where prosthetic elements and other customized products can be individually manufactured. It is already used in dental prosthetics on a large scale (Figure 5).

There are numerous examples of various industrial sectors where parts and components are produced using 3D printing technology (Table 3). It is widely used in automotive, aerospace, healthcare, architecture, and jewelry industries. Its ability to produce customized, complex designs and prototypes rapidly has revolutionized manufacturing processes. In the jewelry sector, AM methods such as SLS and DMLS enable the creation of intricate, high-quality pieces from precious metals that traditional methods cannot easily replicate.

The development of 3D printing technology translates into market trends and economic aspects. The global 3D printing market has shown substantial growth. As of 2023, the market size has reached USD 24 billion and is expected to grow significantly due to further technological advancements and the increasing demand for 3D printing technology in various industries (Table 3).

In Europe, the market is expanding, with Germany leading in innovation and application. The Germany 3D printing market generated a revenue of USD 2210.3 million in 2023 and is expected to reach USD 10,235.3 million by 2030 [141].

Similarly, the U.S. market is expected to see rapid growth, fueled by government investment and technological advancements. The North America 3D printing market size is expected to reach USD 16.59 Billion by 2032 [145].

The 3D printing market in China is expected to reach a projected revenue of USD 7907.2 million by 2030. A compound annual growth rate of 27.6% is expected of China’s 3D printing market from 2024 to 2030 [209] (Figure 13 and Figure 14).

It is possible to use recycled materials to produce AM products. In turn, the method of producing AM products itself, by definition, does not generate waste because it uses the entire input material. Due to these features, it is a technology that fits perfectly into the principles of the circular economy. Three-dimensional printing technology, although consistent with the principles of a closed-loop economy, uses energy related to the production and operation of devices, which may come from non-renewable sources. Metal recycling, however, is more efficient, with metals like aluminum being nearly 100% recyclable. Although 3D printing is perceived as an environmentally friendly technology, it poses major ecological challenges due to the specific properties of some materials used in production, the need for the final processing of products, and the need to clean equipment from material residues. Data show that currently only about 30% of 3D-printed waste is recycled. As in other industries, the issue of recycling and the introduction of a closed-loop economy require urgent systemic solutions. Due to trends in environmental protection and the assumptions of Industry 4.0, and especially 5.0, the prospects for implementing ecological solutions seem to be good.

Additive manufacturing is an important element of Industry 5.0, in particular filling the growing demand for personalized products. It is also highly resistant to crises in the supply of materials, because it largely uses recycled materials. Three-dimensional printing technology has revolutionized traditional technological methods in various industries. It illustrates potential further development towards versatility and increasing innovation in the economy. The ongoing advancements in AM technology promise continued growth and innovation, with significant potential to impact various sectors positively. As the technology evolves, balancing its benefits with environmental considerations will be crucial to ensuring a sustainable future.

Advanced 3D printing technologies controlled by human-defined algorithms are the essence of the interpretation of the idea of Industry 5.0 and new industrial transformation. This convergence is not merely a technical upgrade but a profound evolution in how we conceptualize and execute manufacturing. Three-dimensional printing, as a cornerstone of Industry 5.0, embodies the very principles of this new industrial paradigm: customization, sustainability, and resilience. The technology’s ability to create complex, bespoke products on demand reduces waste and shortens supply chains, perfectly aligning with the circular economy goals of Industry 5.0. By integrating recycled materials and minimizing the carbon footprint, 3D printing not only addresses environmental concerns but also redefines the possibilities of material usage. The human factor, a hallmark of Industry 5.0, finds its most impactful expression through 3D printing. This technology democratizes manufacturing, bringing the power of production to individuals and small businesses, thus fostering innovation at the grassroots level. As we embrace this technology, we are not only advancing industrial capabilities but also empowering creativity and adaptability in an ever-evolving market. Looking forward, the synergy between Industry 5.0 and 3D printing promises to unlock unprecedented opportunities. Imagine a world where manufacturing is not just efficient and sustainable but also deeply personalized, where every product is a unique reflection of individual needs and environmental consciousness. The integration of 3D printing technology with Industry 5.0 is not only a technological evolution, but a long-term revolutionary change in the way products are manufactured, which is particularly reflected in the response to individual needs. It heralds a future where industrial processes are seamlessly intertwined with human values and environmental stewardship, creating a new era of manufacturing that is both advanced and deeply humane. This is the promise of Industry 5.0—a promise that is being realized with every layer of material deposited, every design brought to life, and every step toward a more sustainable and equitable future.

## 8. Conclusions

The research and analysis conducted in this paper lead to the following main conclusions characterizing the basic features, advantages, and disadvantages, as well as the applications of 3D printing.

1. Three-dimensional printing technology is an integral part of Industry 4.0 and 5.0. In particular, it meets the assumptions of Industry 5.0 due to cooperation with a human who designs individual solutions using advanced software and artificial intelligence.
2. Three-dimensional printing shortens supply chains, saves materials, and solves the problem of obtaining parts without excessive storage. Shortening supply chains and the possibility of using recycled materials are beneficial aspects of 3D printing technology due to material shortages resulting from geopolitical conditions.
3. The adaptation of 3D printing in a closed-loop economy will require the systemic development of recycling rules and the analysis of the possibilities of filament recycling. Currently, only 30% of the material waste from 3D printing is recycled.
4. AM technology will work as a complement to classical technologies, especially when combined with polymer injection molding processes.
5. Three-dimensional printing is primarily profitable for small-batch production and the production of individual personalized products. This corresponds to the assumptions of Industry 5.0 regarding the personalization of products.
6. Prospectively, the greatest demand for 3D printing products will occur in the medical and prosthetic (dental) industries, which already widely use this technology.
7. Industries such as automotive and aviation industries successfully use 3D printing.
8. Due to its limitations, 3D printing will not replace classic technologies producing large-sized elements and large-scale products.
9. Three-dimensional printing technology uses a large number of various materials but has material limitations resulting from the use of high temperatures, which affect the structure and properties of the products. The most common materials used in 3D printing technology are polymers.

## Figures and Tables

**Table 1:** Basic differences between Industry 4.0 and 5.0.

Industry 4.0	Industry 5.0
implementation of automatic devices and digital solutions	implementation of automatic devices and digital solutions
linking the operation of machines with IT systems	combining work performed by people and machines so that they complement each other
automation and digitization of industrial processes	cooperation between people and machines, making complex decisions
using data to make decisions and increase efficiency	more flexible and personalized production process
focus on digitization and connection of industrial production systems using advanced technologies such as the Internet of Things or cloud computing	focusing on cooperation between people and robots, combining human abilities and knowledge with the efficiency and precision of machines
technological tasks are performed by machines, processes are automated	strengthening the role of employees in the production process
emphasizes mass customization and flexible production, which allows products to be tailored to customer needs	promoting highly personalized production to order; articles are produced in a very individualized way, in accordance with the customer’s preferences
technologies such as the Internet of Things, big data, artificial intelligence, virtual reality, and cloud computing dominate; their task is to automate processes, analyze large amounts of data, and support predictive decision-making	in addition to technology, there are advances in collaborative robots (cobots), exoskeletons, augmented reality, and advanced artificial intelligence systems that enable close interaction between humans and machines
striving to increase efficiency and reduce energy consumption using automation and cutting-edge technologies	places greater emphasis on the use of renewable energy sources and the design of energy-efficient systems to achieve a more sustainable production process and reduce the carbon footprint

**Table 2:** Main 3D printing technology classification.

Main 3D Printing Technology	Varieties of Technology	Process Characterization
Vat photopolymerization Vat photopolymerization uses a vat of liquid photopolymer resin from which the model is built layer-by-layer. Ultraviolet (UV) light is used to harden the resin when required, while the platform moves the object being created downwards after each new layer has cured.	Stereolithography (SLA)	Laser to trace each layer individually.
	Digital Light Processing (DLP)	Irradiates each layer all at once using a projector, resulting in faster print times.
	Liquid Crystal Display (LCD)	Uses an LCD mask to control light exposure, potentially allowing for larger build volumes.
	Continuous Liquid Interface Production (CLIP)	Compared to SLA, DLP significantly reduces printing time by solidifying an entire layer at once.
	PolyJet	Print heads spray successive layers of liquid light-curing photopolymer onto the build platform, which then hardens under the influence of ultraviolet (UV) light.
	Computed Axial Lithography, CAL	This process allows you to create the desired 3D structure in one printing step.
Directed Energy Deposition (DED) It is a 3D printing method which uses a focused energy source, such as a plasma arc, laser, or electron beam to melt a material which is simultaneously deposited by a nozzle. Most frequently used for metals in powder or as a wire.	Laser Metal Deposition (LMD)	A laser is used as an energy source to melt metal powders.
	Cold Spray (CS)	CS produces parts with a very high density, >99%, due to the very high velocity imposed on the particles.
Power Bed Fusion (PBF) Any powder-based materials can be used in PBF technology. Thermal energy is used to selectively combine the powder, which in various versions of PBF technology is obtained from various sources. It may be a laser pulse, an electron beam, or a heated print head. The material granules can be sintered or fused, depending on the heat intensity level.	Laser Powder Bed Fusion (SLM)	Laser heat is used to fuse metallic powders.
	Electron Beam Melting (EBM)	Melting with an electron beam.
	Selective Laser Sintering (SLS)	Selective hot sintering.
	Direct Metal Laser Melting (DMLM)	Direct laser melting of metals.
	Directed Metal Laser Sintering (DMLS)	Directed laser sintering of metals.
Material Extrusion (FDM) In this technology, the material is extruded through a heated nozzle. The process involves extruding a plastic thread in accordance with a computer-defined program for shaping a three-dimensional object. The dimensional accuracy of the process is: ±0.5% (lower limit ±0.5 mm).	Fused Deposition Modeling (FDM)	It is an extrusion-based method where filament, produced by hot-melt extrusion (HME), is heated within the head of the printer cartridge and extruded through a nozzle onto a build platform.
	Direct Ink Writing (DIW)	Direct Ink Writing (DIW) is an extrusion-based additive manufacturing method heavily utilized in meso- and micro-scales.
Binder Jetting (BJ) The print head selectively sprays a liquid bonding agent onto a thin layer of building material, layer-by-layer, according to the prepared bitmap. After the printing process, the manufactured products are sintered in a high-temperature furnace.		The process involves applying layers of metal or ceramic powder to the surface of the worktable. Then, a special, numerically controlled print head applies a liquid binder, creating three-dimensional units called voxels. During heating and sintering, the binder, usually based on photo polymers, evaporates in the later stages of the process.
Material Jetting (MJ) In the spraying process, molten material droplets are deposited on the 3D printer’s working platform and then hardened with ultraviolet light or heat. In this way, three-dimensional objects are formed layer-by-layer.	Poly Jet	It uses liquid photopolymer resins hardened layer-by-layer with UV light. Piezoel-electric heads apply a layer of liquid material to the work table, and then each layer is automatically exposed to a UV light head. After hardening, another layer of resin is applied.
	NanoParticle Jetting (NPJ)	Compared to other technologies, it offers accuracy, high resolution and design freedom thanks to the easily soluble support material. NanoParticle Jetting produces parts by ejecting thousands of nanoparticles from ink nozzles in ultra-thin layers. The deposited nanoparticles vary in size and shape and are randomly distributed on the working platform, creating highly packed structures.
	Drop-On-Demand (DOD)	Drop on Demand (DOD) involves placing molten drops of material onto the surface until a finished object is obtained.
Sheet Lamination (SL) The sheet lamination (SL) manufacturing technique, also known as laminated object manufacturing (LOM), consists of superpositioning several layers of material composed of foil to manufacture an object. These sheets of material are glued together layer-by-layer and cut into shape using a knife or with laser cutting.	Matt Lamination	Matte laminate has a “natural” appearance with lower contrast than other products, which ensures a velvety texture of the products.
	Glossy Lamination	The product is shiny, resistant to dirt, dust and fingerprints.
	Velvet Lamination	Velvet lamination involves coating plain paper with a layer of plastic. As a result, the product has a softer outer surface and higher quality.

**Table 3:** Advantages and disadvantages of 3D printing technology.

Technology 3D	Advantages	Disadvantages	Materials and Applications
Vat photopolymerization [60]	Ability to produce final details with high dimensional accuracy, the smoothest finish surface, and dimensional tolerances are below 0.05 mm. Finished models can be painted, moderately fast. Economical for producing small quantities of parts.	The method requires the use of supports, materials for production are expensive, the processing of products is long and complicated, the resin is toxic, and its mixing with IPA makes it even more dangerous, the liquid must be secured and sent for disposal to a specialized company, the waste is not suitable for recycling and is difficult to manage, prints are weakest in the vertical direction of the structure due to the anisotropy of the material properties and due to the additive layer method, the laser must be calibrated periodically, the thickness of the layers may be different for different resins, the work table is covered with a layer of uncured resin that must be removed, which affects the costs and extends the production time of the detail.	Materials: main components of photopolymerization prepolymers or oligomers, monomers, and photo initiators. Oligomers such as acrylates and epoxy resins are the main components of the photopolymer, which determine the physical properties of the SLA product, synthetic hydrogel monomers are polyethylene glycol (PEG), polyacrylic acid, polylactic acid, polyglycolic acid, or poly (lactic-co-glycolic acid). Bio resins composed of natural and synthetic polymers, such as polyethylene glycol-conjugated chitosan and the combination of alginate with methacrylate gelatin (GelMA), are also popular choices for VP-based bioprinting. The bio resins employed in VP-based bioprinting are primarily composed of solvent water, photoactive monomers or oligomers, and PI. Applications: Medicine and dentistry production of implants and various prototypes; photopolymerization is successfully used in medical modeling, which allows the creation of accurate 3D models of various anatomical areas of the patient based on data from computer scans. Vat photopolymerization is also particularly suitable for the production of small components made of technical ceramics.
Direct Energy Deposition (DED) [61]	Possibility to create very large elements, high production speed, creation of high-density details, possibility to repair damaged details, possibility to apply coatings resistant to abrasion, corrosion, etc., adding metal elements to existing parts, producing details from several materials in one process.	Parts require heat treatment to remove residual stresses, residual stresses may lead to deformation and damage to printed elements, low surface quality, and complicated internal channels are an additional challenge.	Materials: Spherical metal powder with a gradation of 45–150 µm or wire with a diameter of 1 to 3 mm is used, typically used to work on metal parts; this process can also be used with polymers and ceramics, almost any weldable metal can be additively manufactured using DED, including aluminum, Inconel, niobium, tantalum, titanium, and titanium alloys, tungsten, stainless steel 316L, 304, 15-5PH, 17-4PH, steels H13 and 1.2709; nickel alloys—Inconel 625, Inconel 718, Hastealloy X, Waspalloy; stopy tytanu: Ti6Al4V ELI; cobalt alloys: Stellite 6, Stellite 12, Stellite 21. Applications: DED can be used to fabricate parts but is generally used for repair or to add material to existing components, and applications for DED fall into three categories; near-net-shape parts, feature additions, and repair.
Power Bed Fusion (PBF) [62]	Production of elements with complicated and complex geometry, maintaining high strength parameters of parts while reducing their weight. Easy removal of unbaked powder. High quality of the surface of printed elements. Possibility of producing elements with fine details, high density details, and construction of cooling channels with a complicated course.	After the printing process, the parts require heat treatment to remove residual stresses, which may lead to deformation and damage to the printed elements, complicated repair of damaged parts, necessity to use support structures.	Materials: Common metals and polymers, metal powders with precisely defined morphology, and particle size-aluminum alloys, nickel alloys, stainless steels, titanium, cobalt and copper alloys, polymers such as SHS, nylon DMLS, SLS, and SLM: Applications: Medicine: L-PBF enables the creation of implants, prostheses, surgical tools, and other highly precise medical components. Automotive: L-PBF 3D printing is used to produce custom automotive parts, exhaust systems, turbochargers, and other components. It is one of the most popular 3D printing techniques used in industrial additive manufacturing (AM).
Binder Jetting (BJT/BJ3DP) [63]	Low costs, high repeatability of prints, allows create large prints; parts do not have residual stresses, very high printing speed; does not require the use of supporting structures; in the printing process, no heat source is used to combine the metal powder. No build platform is required; economically viable.	Dimensional instability of printed objects, parts require an additional sintering process or infiltration in the furnace to improve their mechanical properties, high porosity of prints even after the sintering process, shrinkage of parts, porosity reduces the strength properties of prints.	Materials: Sand and metal powders, ceramics, metals (316L stainless steel, Inconel 625, titanium 6Al-4V, aluminum (various alloys), copper, tool steel, maraging steel, cobalt chrome, nickel alloys, precious metals, and various plastics. Applications: For the production of full-color prototypes, low-cost metal parts, the production of large cores and sandblasting casting molds, aerospace components, chemical processing equipment, turbine blades, heat exchangers, rocket engine components, aerospace components, medical implants, sporting goods, car parts, radiators, electronic housings, heat exchangers, electrical components, radiators, cutting tools, dies, molds, aerospace components, defense applications, joint replacement, dental implants, coprocessing equipment, oil and gas components, jewelry, electronic components, and medical devices (limited applications).
Material Extrusion (FDM [64])	The cost of printing compared to other technologies is relatively low, availability of various materials, ease of subsequent processing of the product, possibility of changing the material during printing to another one, short prototyping time.	Lower print accuracy compared to other technologies, during complex prints it is difficult to remove the support material, lower durability compared to other technologies, print layers are often very visible to the human eye.	Materials: thermoplastic polymers. Materials used in FDM processing include polycaprolactone (PCL), polypropylene (PP), polyethylene (PE), polybutylene terephthalate (PBT), acrylonitrile butadiene styrene (ABS), wood, nylon, metals, carbon fiber, graphene-doped PLA, etc. Polymer nanocomposites, hydrogels, alloys and pure metals with melting points up to 700 degrees Celsius. The most common FDM 3D printing materials are PLA, ABS, PET, nylon, TPU (Flexible), PC and their various blends. Applications: FDM is used in aerospace and automotive industries. Fused deposition modeling or FDM is the most popular method of 3D printing for pharmaceuticals.
Material Jetting (MJ) [65]	It is one of the fastest as well as one of the most accurate 3D printing technologies, developed different types of material jetting for different applications, integrating multiple material parts and colors in one single printing, and can be used for a combination of multiple materials.	Requires supports. After printing, support materials are dissolved or removed manually, leaving behind a clean, smooth object. The printing materials must have the right flow properties to ensure precision without compromising strength, the environment and machine need to maintain optimal temperature conditions to avoid material expansion or contraction. It can be more expensive compared to other 3D printing techniques.	Materials: The most commonly used are PLA, ABS, and PET-G. Applications: It is used by a multitude of industries such as automakers, design firms, art studios, or medical organizations to create reliable prototypes with a high level of accuracy. NanoParticle Jetting (NPJ) is the best option when you need to manufacture multiple small parts at once. Drop-On Demand (DOD) is the preferred choice of the jewelry industry. Material jetting is a printing process with a high level of accuracy, which makes it popular for highly realistic prototypes, injection molds, investment castings, and medical devices.
Laminating Sheets or Laminated Object Manufacturing (LOM) [66]	Relatively economical in terms of material costs, the process is usually fast and effective, high precision of workmanship, the cheapest operation due to the lowest costs of obtaining appropriate paper, high accuracy, and very fast printing process, relatively low prices of materials for creating models.	The product requires cleaning and a large amount of waste that cannot be reused.	Materials: A variety of materials, metals, adhesive-coated paper, polyester laminate, and plastic. Applications: Manufacturing industry, for creating prototypes, tools, structural elements, and spare parts. In the medical industry, for the production of anatomical models, creating prostheses, and implants. Art and design, architecture, and education, for business cards, brochures, paper bags, boxes, flyers, posters, books, roll-up banners, and other marketing and promotional materials.

**Table 4:** Three-dimensional printing methods used to produce parts from metals and polymers.

Type of Material	3D Printing Method
Metal printing	SLM (Selective Laser Melting) LPBF (Laser Powder Bed Fusion) DMLS (Direct Metal Laser Melting) FDM (Fused Deposition Modeling) SLS (Selective Laser Sintering)
Polymer printing	SLA (Stereolithography) DLP (Digital Light Processing) MJF (Multi Jet Fusion) FDM (Fused Deposition Modeling)

**Table 5:** Three-dimensional printing process parameters.

3D Printing Technology	Technology Parameters
Vat Photopolymerization	Vertical build rate: up to 18 mm per hour (material and layer thickness dependent) Materials: Poly(ethylene glycol) diacrylate (PEGDA), Poly(ethylene glycol) dimethyacrylate (PEGDMA), other diacrylate or dimethyacrylate functionalized polymers, and ceramic suspensions., Speed DLP/SLA 20–36 mm/h average, maximum 720 mm/s, SLA 48 mm/s, maximum 60 mm/s. In systems using the radical cross-linking mechanism, difficulties include not only sensitivity to oxygen inhibition, but also relatively high polymerization shrinkage of about 20%; printing parameters (e.g., single layer thickness, exposure time) are selected individually for each operation: the layer height should not exceed 75% of the nozzle thickness, the average layer thickness can be 50–100 µm, and in operation, the layer thickness reached 306 μm.
Direct Energy Deposition (DED)	Electron beam DED has the highest rate of up to 9 kg/h (20 lbs/h). Laser- and powder-based DED systems deposit material at the rate of 3 kg/h (6.5 lbs/h). Powder bed fusion systems have the lowest rate with deposition rates below 0.2 kg/h (0.44 lbs/h). The typical cooling rates are in the range of 10^2^ to 10^4^ K/s. Defined heating and cooling rates are important parameters for DSC measurements. International standards recommend a heating rate of 10 K/min or 20 K/min (ISO 11357, DIN 53765, ASTM E 793, ASTM E 794) when striving for thermodynamic equilibrium. Typical power values of our DED machines range between 5 and 20 kW, varying with material, deposition rate, and geometry. Typical layer thicknesses of 0.25 mm to 0.5 mm are recommended. The cooling times for materials are very fast, at around 1000–5000 °C per second.
Power Bed Fusion (PBF)	The power of the lasers used are 200–500 watts and the electron-beams are 1000–6000 watts. The particle size required for L-PBF ranges between 15 and 60 microns; E-PBF uses larger particles, between 45 and 105 microns. The deposition rate of the powder-feed/-bed technology is extremely low, typically around 10 g/min, and typically, a 0.1mm thick material is spread over the build platform. The standard film thickness for a standard powder coating for optimum mechanicals is between 60 and 80 microns. A classical active layer can be thawed to a depth of approximately 2–8 cm, and the thickest AL reaches over 20 m. L-PBF systems print most materials at speeds of 10–50 cc/h, while E-PBF prints at 50–90 cc/h (highly material dependent).
Material Extrusion (ME)	Alloys are extruded at speeds between 12 and 65 mm/s; the rate depends on the capacity and power of the equipment employed. Print speeds generally fall between 100 and 150 mm/s. Typical layer thickness varies from 0.178 mm to 0.356 mm. For great detail and layer-to-layer bonding, the thickness of each layer is kept constant between 0.1 mm and 0.2 mm.
Binder Jetting (BJ)	A typical resolution is 35 μm. Minimum feature sizes as low as 0.1 mm can be printed. Binder jetting typically reaches a dimensional tolerance of ±0.5% of the outer dimension and at best ±50 μm. Binder jetting also allows for complex geometries, does not require support structures, and has a dimensional accuracy of ±0.2 mm. In binder jetting, the layer thickness typically varies between 15 and 300 μm. The selection of a layer thickness is based on the size distribution of the powder, which, in turn, defines the resolution of the process along the build direction. Binder jetting also allows for complex geometries, does not require support structures, and has a dimensional accuracy of ±0.2 mm. The recommended minimum wall thickness for parts produced via binder jetting is 2.0 mm.
Material Jetting	Inkjet speed: 4000 drops of binder per nozzle per second. Build speed: up to ~200 cm^3^/min of parts. MJ technology supports dimensional tolerances in the range of ±300 µm (0.3 mm). A Hydro Jetting Machine is used for pressures ranging from 140 bar (2000 PSI) to 1400 bar (20,000 PSI). For material jetting, the typical layer height is 16–32 microns. Material jetting has a dimensional accuracy of ±0.1%. It has a typical lower limit of ±0.1 mm.
Sheet Lamination	The brow lamination process itself does not take very long, only around 15–20 min. It is recommended to adjust the temperature of the laminator as per below, given indicative temperature settings: 80 Microns −110 °C. 125 Microns −140 °C. 175 Microns −160 °C. It can take 24 to 48 h for the lamination and drying process. The laminate needs a minimum of 48 h to acclimate, but a full three days is best. The standard thickness for laminating pouches is 3 mil, 5 mil, 7 mil, and 10 mil. The thickness of the transformer core laminations is usually in the order of 0.25 mm to 0.5 mm. Laminate ranges from 6 mm to 12 mm and, as a rule, it should not be less than 8 mm. If, however, budget is an issue and if your subfloor is level and debris-free, you might be able to get away with 7 mm; the standard panel thickness is 10 mm, 13 mm, or 18 mm, with variants from 6 mm to 25 mm available for special applications.
